# Single-cell, single-mRNA analysis of *Ccnb1* promoter regulation

**DOI:** 10.1038/s41598-017-02240-y

**Published:** 2017-05-18

**Authors:** Nidhi Vishnoi, Jie Yao

**Affiliations:** Department of Cell Biology, Yale University School of Medicine, New Haven, CT 06510 USA

## Abstract

Promoter activation drives gene transcriptional output. Here we report generating site-specifically integrated single-copy promoter transgenes and measuring their expression to indicate promoter activities at single-mRNA level. mRNA counts, Pol II density and Pol II firing rates of the *Ccnb1* promoter transgene resembled those of the native *Ccnb1* gene both among asynchronous cells and during the cell cycle. We observed distinct activation states of the *Ccnb1* promoter among G1 and G2/M cells, suggesting cell cycle-independent origin of cell-to-cell variation in *Ccnb1* promoter activation. Expressing a dominant-negative mutant of NF-YA, a key transcriptional activator of the *Ccnb1* promoter, increased its “OFF”/“ON” time ratios but did not alter Pol II firing rates during the “ON” period. Furthermore, comparing H3K4me2 and H3K79me2 levels at the *Ccnb1* promoter transgene and the native *Ccnb1* gene indicated that the enrichment of these two active histone marks did not predispose higher transcriptional activities. In summary, this experimental system enables bridging transcription imaging with molecular analysis to provide novel insights into eukaryotic transcriptional regulation.

## Introduction

Single cell imaging has become a powerful approach in probing and understanding the mechanisms of gene transcription *in vivo* ^1–5^. Earlier work studied the recruitment kinetics of transcription factors and RNA polymerases in living cells^6–8^ and measured the residence times of transcription factors at gene loci^6, 9, 10^, rates of transcriptional elongation^11–13^, etc. Coupling live cell imaging with mathematical modeling provided reliable measurements on transcription kinetics of single-copy endogenous genes in yeast and mammalian cells^14–17^. Improved single molecule tracking enabled analyzing the diffusion and binding kinetics of individual transcription factor molecules in living mammalian cells^18–20^. Given these significant advancements in single cell transcription imaging, an important task is to improve our fundamental understandings on the regulatory mechanisms of specific genes or promoters *in vivo*.

Single-mRNA detection has provided an invaluable approach to directly and precisely measure transcriptional output *in vivo* ^21–23^. The Singer lab generated transgenic mouse with the endogenous *β*-*actin* gene tagged with 24X MS2 repeats and analysed its transcriptional activation kinetics during serum response^15^. In another approach, single-copy transgenes driven by the *CMV* promoter and the *CCND1* promoter were generated by site-specific DNA recombination in HEK293 human embryonic kidney cells that allowed analysing transcription kinetics at the single-mRNA level^24^. Nonetheless, given that the mammalian genome is pervasively transcribed^25^, this work did not address to what extent transgene expression reflected promoter activation or mimicked the endogenous gene. In this paper, we report an experimental system to measure transcriptional output from a single-copy transgene driven by the cell-cycle regulated *Ccnb1* promoter.

Cyclins are an important group of highly-conserved proteins that interact with cyclin-dependent kinases and regulate cell cycle progression^26^. The B-type cyclins are of particular interest because their expression levels are elevated in G2/M to promote mitotic entry^27, 28^. In mammalian cells, increased expression of the *Ccnb1* gene in G2/M resulted from increased gene transcription and in some cases, increased mRNA stability^27, 29–32^. Although previous studies have identified several transcription factors and DNA elements regulating the *Ccnb1* promoter^33–36^, how the *Ccnb1* promoter is transcribed at the single cell level and during the cell cycle is not well understood. Single cell analysis of *Ccnb1* promoter activation will avoid averages over cell populations or over cell cycle stages, and thereby advance our understandings on *Ccnb1* promoter regulation *in vivo*.

In this study, we have engineered mouse C2C12 myoblasts by integrating a single-copy transgene driven by the mouse *Ccnb1* promoter into an identified genomic locus on chromosome 19. The transgene mRNA contains 24X MS2 repeats allowing detection by single molecule RNA FISH or live cell imaging. Expression of a No-promoter transgene integrated at the same locus was over one order of magnitude lower than that of the *Ccnb1* promoter transgene, supporting that transgene expression predominantly resulted from *Ccnb1* promoter activation. Furthermore, mRNA counts and Pol II densities of the *Ccnb1* promoter transgene recapitulated those of the native *Ccnb1* gene during the cell cycle. Using this system, we observed distinct *Ccnb1* promoter activation states at the single cell level and compared active histone modification levels at the *Ccnb1* promoter transgene and the native *Ccnb1* gene. Furthermore, we found that a key transcription activator NF-Y controls the “OFF”/“ON” time ratios of the *Ccnb1* promoter. Our results demonstrate that the single-copy promoter transgene approach can be applied to quantify promoter activities at the single cell level and to measure regulated changes in promoter activities with single-mRNA sensitivity.

## Results

### Generating and characterizing single-copy promoter transgenes

**Figure 1:**
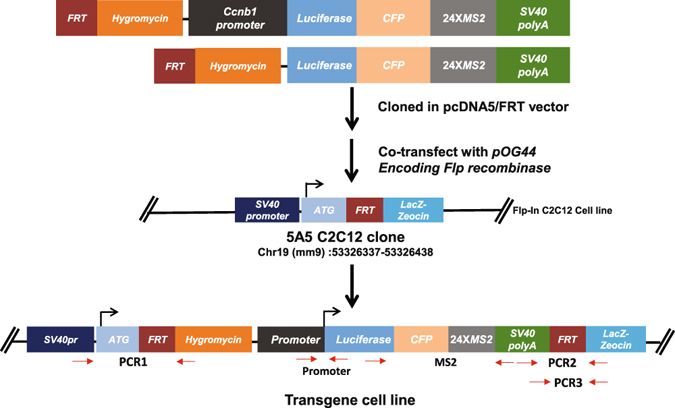
Generating a C2C12 cell system that allows integration of single-copy promoter transgenes at an identified locus. Diagrams of constructs and the transgene locus are shown. Mouse *Ccnb1* promoter (~2.8 Kb) was cloned upstream of the *Luciferase* coding region in the *pcDNA5*/*FRT* vector. 24X MS2 repeat sequence was cloned downstream of the *Luciferase* coding region and before the SV40 late poly(A) signal. As a control, we cloned a vector containing the *Luc*-*MS2* transgene without a promoter. The constructs were then cotransfected with pOG44 (encoding Flp recombinase) into the C2C12 Flp-In cell clone 5A5, which has a single copy insertion of the *pFRT/LacZeo* vector at mouse chromosome 19 (~53 Mb). Locations of primers used to validate the correct integration of transgenes are shown by red arrows.

Motivated by studying transcriptional regulation during myogenic differentiation, we have generated and characterized a mouse C2C12 myoblast cell clone containing a single FRT site that allows site-specific integration of transgenes (Fig. 1). We mapped the FRT site insertion locus to the first intron of a non-coding RNA gene (*1700001K23Rik*) in mouse chromosome 19 (Supplementary Fig. S1). Next, we integrated a single-copy *Ccnb1* promoter transgene or a No-promoter transgene into this FRT site in mouse C2C12 myoblasts (Fig. 1). These transgenes contain the *Luciferase* and cyan fluorescent protein (*CFP*) coding region, a 24X MS2 cassette at the 3′-untranslated region (UTR) that allows single mRNA detection^15, 37^ and an SV40 late polyadenylation signal. The *Ccnb1* promoter transgene contains around 2.8 kb DNA sequences upstream of the transcription start site of the *Ccnb1* gene (Supplementary Fig. S2). We verified correct integration and the integrity of transgenes by PCR using primers spanning the two FRT sites and different regions of transgenes (Supplementary Fig. S2). We carried out combined MS2-RNA FISH and DNA FISH using probes targeting a BAC probe targeting the chromosome 19 insertion site (Supplementary Fig. S1). We found that DNA FISH signals from the chromosome 19 insertion site were colocalized with the transcription site (TS) detected by MS2 RNA FISH (Supplementary Fig. S1), thus confirming that the integrated single-copy *Ccnb1* promoter transgene was transcriptionally active.

**Figure 2:**
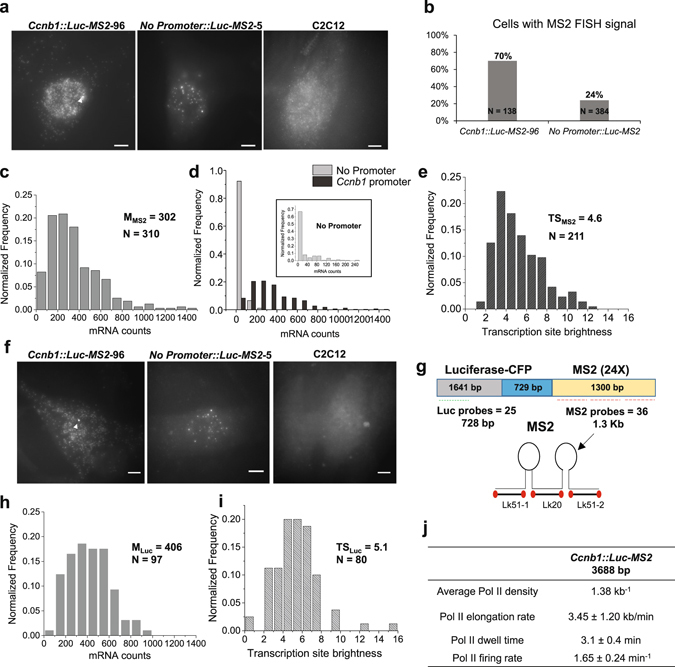
Quantifying mRNA counts and TS brightness from cell clones containing *Ccnb1*::*Luc*-*MS2* and *No Promoter*::*Luc*-*MS2* transgenes. (**a**) Representative single molecule RNA FISH images of *Ccnb1*::*Luc*-*MS2* (clone 96), *No Promoter*::*Luc*-*MS2* (clone 5) and C2C12 cells using MS2 FISH probes. (**b**) Fractions of cells expressing the *Ccnb1*::*Luc*-*MS2* transgene or *No Promoter*::*Luc*-*MS2* transgene (average of three cell clones). (**c**) Histograms of mRNA counts of the *Ccnb1*::*Luc*-*MS2* transgene measured by FISH analysis using MS2 probes. M_MS2_ indicates the median mRNA count measured by MS2 FISH. N indicates the measured cell number. (**d**) Histograms of transgene mRNA counts in *Ccnb1*::*Luc*-*MS2* clone 96 (dark grey) and *No Promoter*::*Luc*-*MS2* cell clones (light grey, average of three cell clones). (Inset) Histograms of mRNA counts in *No Promoter*::*Luc*-*MS2* cell clones with a bin of 20. The fraction of *No Promoter*::*Luc*-*MS2* cells with zero mRNA counts was included in the histogram plot. (**e**) Histograms of TS brightness of the *Ccnb1*::*Luc*-*MS2* transgene measured by FISH using MS2 probes. TS_MS2_ indicates the median TS brightness measured by MS2 FISH. N indicates the measured TS number. In panels (c–e), FISH images from three experiments were analysed. (**f**) Representative single molecule RNA FISH images of *Ccnb1*::*Luc*-*MS2* (clone 96), *No Promoter*::*Luc*-*MS2* (clone 5) and C2C12 cells using FISH probes targeting the 5′-*Luciferase* coding region. (**g**) Schematic diagram depicting the *Ccnb1*::*Luc*-*MS2* transgene and positions of FISH probes (not to scale). A mix of twenty-five 5′-labelled probes were used to detect the 5′-portion of the *Luciferase* coding region of *Luc*-*MS2* mRNA. (**h**,**i**) Histograms of mRNA counts and TS brightness (indicating nascent transcript counts) of the *Ccnb1*::*Luc*-*MS2* transgene measured by FISH analysis using *Luciferase* probes. M_Luc_ and TS_Luc_ indicate the median mRNA count and the median TS brightness measured by *Luciferase* FISH, respectively. N indicates measured cell number and TS number, respectively. FISH images from one experiment were analysed. (**j**) Table summarizing estimated average RNA Pol II density, Pol II elongation rate, dwell time and firing rate at the *Ccnb1*::*Luc*-*MS2* transgene. In panels a and f, white arrowheads indicate the TSs. All scale bars: 5 µm.

We measured transgene expression by single molecule RNA FISH (Fig. 2a) using FISH probes targeting the MS2 cassette in the *Ccnb1*::*Luc*-*MS2* cells (clone 96). We found that ~70% of cells expressed the *Ccnb1*::*Luc*-*MS2* transgene and ~24% of cells expressed the *No promoter*::*Luc*-*MS2* transgene (Fig. 2b). Expression of the *No promoter*::*Luc*-*MS2* transgene likely resulted from the weak expression at the integration site or read-through from the upstream *SV40*::*Hygromycin* gene. mRNA counts among cells expressing the *Ccnb1*::*Luc*-*MS2* transgene approximately followed Gaussian distribution with the median value of 302 (Fig. 2c). In some cells mRNA counts were higher than 1000 (Fig. 2c,d). In contrast, mRNA counts among the majority of cells expressing the *No promoter*::*Luc*-*MS2* transgene were lower than 20 (Fig. 2d, Inset). Therefore, our single molecule RNA FISH data confirmed the RT-qPCR data that *Luciferase* expression from the *Ccnb1*::*Luc*-*MS2* transgene was over 10-fold higher than from the *No promoter*::*Luc*-*MS2* transgene (Supplementary Fig. S2). We measured TS brightness (fluorescence intensity of the TS normalized by the average intensity of an mRNA) of the *Ccnb1*::*Luc*-*MS2* transgene with the median of 4.6 (Fig. 2e). TS brightness had been calculated to measure nascent transcript counts at single-copy genes in yeast^22^ or mammals^38^. We obtained similar results when we analysed another cell clone (*Ccnb1*::*Luc*-*MS2* clone 134) containing the *Ccnb1*::*Luc*-*MS2* transgene (Supplementary Fig. S3). Our analysis thus indicated that the *Ccnb1*::*Luc*-*MS2* transgene was active at the insertion site with low background expression. Notably, this system allows measuring promoter transgene expression without significant interference from background expression of the insertion site.

Next, we performed single molecule RNA FISH using probes targeting the 5′-region of *Luciferase* mRNA (Fig. 2g) to detect the 5′-portion of nascent transcripts (Fig. 2f). Because these probes could detect nascent transcripts at 5′-portion of the *Luc*-*MS2* transgene, TS brightness measured by *Luciferase* RNA FISH was a better indicator of nascent transcript counts. We determined the median nascent transcript counts at the TS to be 5.1 (Fig. 2i) and the average Pol II density on the transgene to be 1.38 kb^−1^ (1.38 Pol II molecules per kb, Fig. 2j). According to the reported transcriptional elongation rates at reporter genes containing MS2 repeats in mammalian cells^13, 17^, we estimated that Pol II elongation rate at the *Ccnb1*::*Luc*-*MS2* transgene was 3.45 ± 1.20 kb/min. Moreover, previous studies revealed that mRNA had a dwell time of ~2.0 min at the 3′-end of the gene for proper 3′-end processing and release^17^. Therefore, Pol II dwell time at a gene locus is the sum of Pol II elongation time and 3′-end processing time, and Pol II firing rate is the ratio of nascent transcript count to Pol II dwell time. Taken together, we estimated Pol II dwell time on the 3.7 kb *Ccnb1* promoter transgene to be 3.1 ± 0.4 min and Pol II firing rate to be 1.65 ± 0.24 min^−1^ (Fig. 2j).

### Transcription of the single-copy *Ccnb1* promoter transgene recapitulated the native *Ccnb1* gene

**Figure 3:**
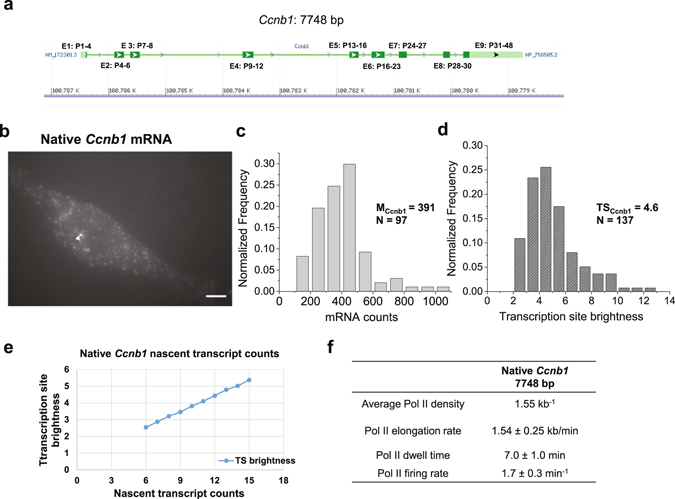
**Quantifying mRNA counts and nascent transcript counts of the native** ***Ccnb1*** **gene**. (**a**) An NCBI genome browser view showing exons (green) and introns of the mouse *Ccnb1* gene (7,748 bp). Positions of forty-eight 20-mer RNA FISH probes (P1 to P48) that target *Ccnb1* exons (2,316 bp) are indicated. (**b**) A representative single molecule RNA FISH image of the native *Ccnb1* gene in *Ccnb1*::*Luc*-*MS2* (clone 96) cells. Scale bar: 5 µm. White arrowhead indicates the TS. (**c**,**d**) Histograms of measured mRNA counts and TS brightness of the native *Ccnb1* gene. M_Ccnb1_ and TS_Ccnb1_ indicate the median mRNA count and the median TS brightness of the native *Ccnb1* gene measured by FISH analysis using *Ccnb1* exon probes, respectively. N indicates the measured cell number and TS number, respectively. FISH images from one experiment were analysed. (**e**) Plot of calculated TS brightness versus nascent transcript counts according to RNA FISH probe locations along the native *Ccnb1* gene. (**f**) Table summarizing estimated RNA Pol II density, Pol II elongation rate, dwell time and firing rate at the native *Ccnb1* gene.

Next, we designed FISH probes targeting exon regions of the native *Ccnb1* gene (Fig. 3a) and examined its expression by single molecule RNA FISH (Fig. 3b). The median mRNA count of the native *Ccnb1* gene was 391 mRNA per cell (Fig. 3c). The median TS brightness of the native *Ccnb1* gene was measured to be 4.6 (Fig. 3d). We designed FISH probes targeting exons, the majority of which were located at the 3′-half of the *Ccnb1* gene (Fig. 3a). Therefore, we calculated the expected TS brightness versus nascent transcript counts according to FISH probe locations (Fig. 3a,e), assuming that nascent transcripts are evenly distributed across the *Ccnb1* gene. We determined the average nascent transcript counts of the *Ccnb1* gene to be approximately 12 and estimated the average Pol II density on the native *Ccnb1* gene to be 1.55 kb^−1^ (Fig. 3f). We used published transcriptional elongation rates of endogenous *Cyclin* and *Cdk* genes in cultured human cells^39^ to estimate the elongation rate of the native *Ccnb1* gene to be 1.54 ± 0.25 kb/min. Assuming that mRNA 3′-end processing time is also ~2.0 min at the native *Ccnb1* gene, we estimated Pol II dwell time on the native *Ccnb1* gene to be 7.0 ± 1.0 min and Pol II firing rate to be 1.7 ± 0.3 min^−1^ (Fig. 3f). Therefore, Pol II density and Pol II firing rate of the *Ccnb1*::*Luc*-*MS2* transgene (Fig. 2j) recapitulated those of the native *Ccnb1* gene (Fig. 3f). The difference in the total mRNA counts of the *Ccnb1*::*Luc*-*MS2* transgene and the native *Ccnb1* gene could result from two expressing native *Ccnb1* alleles in some cells and from different half-lives of *Luc*-*MS2* mRNA and native *Ccnb1* mRNA (Supplementary Fig. S4). Therefore, we have validated that the *Ccnb1* promoter transgene integrated at an identified locus on mouse chromosome 19 exhibited similar transcriptional levels as the native *Ccnb1* gene.

### Expression of the *Ccnb1* promoter transgene during the cell cycle and during differentiation mimics the native *Ccnb1* gene

*Ccnb1* gene expression and promoter activity increase in the G2/M phase^27, 29, 30^ and decrease during terminal differentiation from myoblasts to myotubes^33, 40^. To determine if the *Ccnb1*::*Luc*-*MS2* transgene responds to regulation by the cell cycle and differentiation similarly as the native *Ccnb1* gene, we measured their expression among cells arrested at different cell cycle stages (Supplementary Fig. S5) and among cells undergoing myogenic differentiation. Indeed, *Ccnb1*::*Luc*-*MS2* transgene expression increased in G2/M phase compared to G1 (Supplementary Fig. S5, Supplementary Results) and decreased as myoblast cells differentiated into multinucleated myotubes (Supplementary Fig. S5), consistent with the native *Ccnb1* gene.

**Figure 4:**
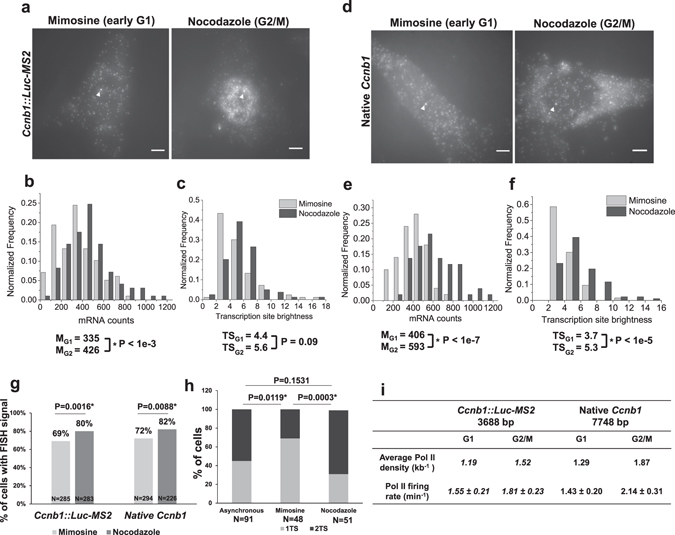
Expression of the *Ccnb1*::*Luc*-*MS2* transgene mimics the native *Ccnb1* gene during the cell cycle. (**a**) Representative single molecule RNA FISH images of the *Ccnb1*::*Luc*-*MS2* transgene using MS2 probes in mimosine-treated or nocodazole-treated cells. Scale bars: 5 µm. (**b**,**c**) Histograms and medians of mRNA counts (**b**) and TS brightness (**c**) of the *Ccnb1*::*Luc*-*MS2* transgene. Measured cell number and TS number were 98 and 83 for mimosine-treated cells, respectively, and were 97 and 79 for nocodazole-treated cells, respectively. (**d**) Representative single molecule RNA FISH images of the native *Ccnb1* gene in mimosine-treated or nocodazole-treated *Ccnb1*::*Luc*-*MS2* (clone 96) cells. In panels a and d, images were taken at 120X magnification. White arrowheads indicate the TSs. Scale bars: 5 µm. (**e**,**f**) Histograms and medians of native *Ccnb1* mRNA counts (**e**) and TS brightness (**f**). Measured cell number and TS number were 50 and 63 for mimosine-treated cells, respectively, and were 51 and 86 for nocodazole-treated cells, respectively. In panels b, e and f, asterisks (*) indicate statistically significant differences in mean values of mRNA counts and TS brightness between mimosine-treated and nocodazole-treated cells determined by student’s t-test (P values are shown in each plot). (**g**) Fractions of cells expressing *Luc*-*MS2* mRNA and native *Ccnb1* mRNA in mimosine-treated and nocodazole-treated cells. (**h**) Fractions of cells with one or two active alleles of the native *Ccnb1* gene in asynchronous, mimosine-treated and nocodazole-treated cells. In panels g and h, asterisks (*) indicate statistically significant differences between the experimental groups determined by Fisher’s exact test. (**i**) Table summarizing estimated RNA Pol II densities and firing rates of the *Ccnb1*::*Luc*-*MS2* transgene and the native *Ccnb1* gene in mimosine-treated and nocodazole-treated cells. Values derived from FISH data using MS2 probes are italicized. FISH images from one successful cell cycle arrest experiment were analysed.

Next, we measured *Ccnb1*::*Luc*-*MS2* transgene expression and native *Ccnb1* gene expression by single molecule RNA FISH in cells arrested at G1 or G2/M (Fig. 4a,d). Median *Luc*-*MS2* mRNA count increased from 335 in G1 cells to 426 in G2/M cells (Fig. 4b). Similarly, median *Ccnb1* mRNA counts increased from 406 in G1 cells to 593 in G2/M cells (Fig. 4e). Thus, mRNA counts of both the *Ccnb1*::*Luc*-*MS2* transgene and the native *Ccnb1* gene increased by 30–50% in G2/M compared to G1 or asynchronous cells (Supplementary Results). Moreover, the fraction of cells expressing the *Ccnb1*::*Luc*-*MS2* transgene increased from 69% in G1 to 80% in G2/M and the fraction of cells expressing native *Ccnb1* mRNA increased from 72% in G1 to 82% in G2/M (Fig. 4g, Supplementary Fig. S6). When we analysed synchronized cells at distinct time points after being released from cell cycle block, we obtained similar results on increased expression of the *Ccnb1*::*Luc*-*MS2* transgene and the native *Ccnb1* gene at G2/M than at G1 (Supplementary Fig. S7, Supplementary Results). Thus, cell cycle progression affects both mRNA counts and the fraction of cells expressing the *Ccnb1*::*Luc*-*MS2* transgene and the native *Ccnb1* gene. We note that single molecule RNA FISH directly measures transcriptional output of promoters and more accurately detects subtle changes in promoter activity due to its sensitivity. In contrast, over 10-fold increase in *Ccnb1* promoter reporter activity during G2/M was reported^29^, which may be due to the non-linear nature of the luciferase reporter assay. We also found that the increase in mRNA counts of both the *Ccnb1*::*Luc*-*MS2* transgene and the native *Ccnb1* gene at G2/M measured by single molecule RNA FISH was more modest than the increase in normalized mRNA levels measured by RT-qPCR (Supplementary Results). Additionally, we found that mRNA counts or the fraction of cells with FISH signals were not significantly different between mimosine and nocodazole-treated cells containing the *No*-*promoter*::*Luc*-*MS2* transgene (Supplementary Fig. S5).

We further compared nascent transcript counts of the *Ccnb1* promoter transgene and the native *Ccnb1* gene during the cell cycle. Median TS brightness of the *Ccnb1*::*Luc*-*MS2* transgene increased from 4.4 in G1 to 5.6 in G2/M (Fig. 4c), and median TS brightness of the native *Ccnb1* gene increased from 3.7 in G1 to 5.3 in G2/M (Fig. 4f). After correcting for gene lengths, we determined that Pol II density increased from 1.19 kb^−1^ in G1 to 1.52 kb^−1^ in G2/M for the *Ccnb1*::*Luc*-*MS2* transgene (which may be underestimated due to using the MS2 FISH probes) and increased from 1.29 kb^−1^ in G1 to 1.87 kb^−1^ in G2/M for the native *Ccnb1* gene (Fig. 4i), using the conversion plot from TS brightness to nascent transcript counts (Fig. 3e). Similarly, using the estimated transcriptional elongation rates (Figs 2j and 3f) and mRNA 3′-end processing times (2 min) of the *Ccnb1*::*Luc*-*MS2* transgene and the native *Ccnb1* gene, we estimated that Pol II firing rate increased from 1.55 ± 0.21 min^−1^ in G1 to 1.81 ± 0.23 min^−1^ in G2/M for the *Ccnb1*::*Luc*-*MS2* transgene and increased from 1.43 ± 0.20 min^−1^ in G1 to 2.14 ± 0.31 min^−1^ in G2/M for the native *Ccnb1* gene (Fig. 4i).

Interestingly, we noticed a higher fraction (~69%) of G2/M-arrested cells contained two active *Ccnb1* alleles than G1-arrested cells (~30%) (Fig. 4h, Supplementary Fig. S6). Likewise, a higher fraction of G2/M-synchronized cells (~37%) contained two active *Ccnb1* alleles than G1-synchronized cells (~22%) (Supplementary Fig. S7). Notably, during both G1 and G2/M, cells with two active *Ccnb1* alleles had significantly higher TS brightness (i.e., Pol II density) but similar mRNA counts as compared to those with one active allele (Supplementary Fig. S8). We suggest that native *Ccnb1* genes rapidly switch between one and two active alleles and increase Pol II density among two-allele cells without affecting mRNA counts. Taken together, our data indicated that: 1) Pol II density and Pol II firing rates of the *Ccnb1*::*Luc*-*MS2* transgene resembled those of the native *Ccnb1* gene in asynchronous cells, G1 cells and G2/M cells. 2) Pol II density and Pol II firing rates of the *Ccnb1*::*Luc*-*MS2* transgene and the native *Ccnb1* gene similarly increased (~30%) in G2/M compared to G1. 3) The fraction of cells containing two active *Ccnb1* alleles was increased in G2/M than in G1. Therefore, we conclude that transcriptional activities at the *Ccnb1*::*Luc*-*MS2* transgene mimic the native *Ccnb1* gene both in asynchronous cells and during the cell cycle. *Ccnb1* promoter transgene is therefore a valuable model to study *Ccnb1* promoter regulation *in vivo*.

### Distinct subcellular localizations of *Luc*-*MS2* mRNA correlate with *Ccnb1* promoter activities at the single cell level

**Figure 5:**
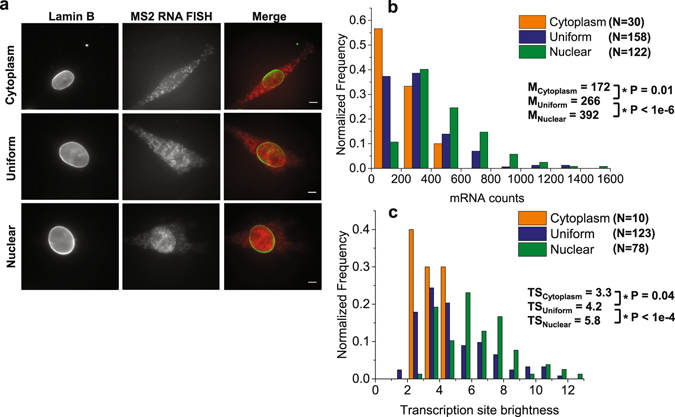
Distinct subcellular localizations of *Luc*-*MS2* mRNA correlate with *Ccnb1* promoter activities. (**a**) Representative single molecule RNA FISH images for *Luc*-*MS2* mRNA (red) and anti-Lamin B immunofluorescence (green) in asynchronous *Ccnb1*::*Luc*-*MS2* (clone 96) cells. Merged images are shown on the right. Cells display cytoplasmic, uniform or nuclear localization of *Luc*-*MS2* mRNA. Scale bars: 5 µm. (**b**,**c**) Histograms and medians of mRNA counts (**b**) and TS brightness (**c**) of the *Ccnb1*::*Luc*-*MS2* transgene among cells exhibiting distinct subcellular mRNA localizations. Measured cell numbers and TS numbers are shown in the plots. Asterisks (*) indicate statistically significant differences determined by student’s t-test (P values are shown in the plots). FISH images from three experiments were analysed.

By single molecule RNA FISH, we observed cell subpopulations exhibiting distinct subcellular localizations of *Luc*-*MS2* mRNA expressed from the *Ccnb1*::*Luc*-*MS2* transgene. We found that *Luc*-*MS2* mRNA molecules were predominantly localized to the nucleus, to the cytoplasm or were uniformly localized to both compartments (Fig. 5a) among asynchronous cells. Cells with nuclear-enriched mRNA exhibited significantly higher mRNA counts and TS brightness than cells with uniformly localized mRNA or cytoplasmic-localized mRNA (Fig. 5b,c). If we assume that Pol II dwell times at the *Ccnb1*::*Luc*-*MS2* transgene are the same, higher TS brightness (indicating nascent transcript counts) is a strong indicator of increased *Ccnb1* promoter activity (i.e., Pol II firing rate) among cells with nuclear-enriched *Luc*-*MS2* mRNA. Furthermore, we confirmed the existence of cell populations differing in *Ccnb1* promoter activity and *Luc*-*MS2* mRNA localization when *Luciferase* probe was used for single molecule RNA FISH (Supplementary Fig. S9) as well as among cells blocked or synchronized at G1 or G2/M (Supplementary Figs S10 and S11, Supplementary Results). Therefore, the observed cell populations with differing *Ccnb1* promoter activity and *Luc*-*MS2* mRNA localizations were not due to RNA FISH probe issues or cell cycle stages, but indicated that the *Ccnb1* promoter displayed a range of transcription states at the single cell level.

The nuclear accumulation of *Luc*-*MS2* mRNA likely resulted from a higher mRNA synthesis rate overriding mRNA nuclear export. Although the native *Ccnb1* gene had similar Pol II densities and Pol II firing rates as the *Ccnb1*::*Luc*-*MS2* transgene, native *Ccnb1* mRNA was predominantly localized in the cytoplasm among all cells examined (Figs 3b and 4d), suggesting that native *Ccnb1* mRNA was exported at a higher rate than the *Luc*-*MS2* mRNA. Given that the *Luc*-*MS2* mRNA was intronless while the native mouse *Ccnb1* gene had 8 introns, this observation was consistent with earlier findings that intron-containing mRNA was exported faster than intronless mRNA^41^. Therefore, single molecule RNA FISH on the intronless *Ccnb1*::*Luc*-*MS2* transgene allowed us to identify variable *Ccnb1* promoter activation states at the single cell level: At a “high” state, mRNA synthesis can override mRNA nuclear export, resulting in nuclear accumulation of *Luc*-*MS2* mRNA; at a “low” state, reduced mRNA synthesis is balanced by mRNA nuclear export and the nuclear accumulation of *Luc*-*MS2* mRNA is diminished.

### Overexpressing a dominant negative mutant of NF-YA modulates *Ccnb1* promoter activation kinetics

How transcription factors regulate promoter activation kinetics is largely unknown. We therefore studied the regulation of the *Ccnb1* promoter at the single cell level by a sequence-specific transcription activator. The CCAAT-binding trimeric transcription factor NF-Y, which is composed of NF-YA, NF-YB and NF-YC subunits^42^, is a key transcription activator of the *Ccnb1* promoter^33, 35, 43, 44^. Indeed, knocking down NF-Y subunits or overexpressing dominant negative mutants of NF-YA inhibited *Ccnb1* gene transcription^33, 45–47^. To understand the role of NF-Y in shaping the transcription kinetics of *Ccnb1*, we analysed transcriptional output of the *Ccnb1* promoter in the presence of a dominant negative NF-YA mutant. Overexpressing the NF-YAm29 mutant that is defective in DNA binding^45^ will sequester NF-YB and NF-YC in defective NF-Y complexes unable to bind to the *Ccnb1* promoter. Accordingly, we generated stable cell lines expressing the NF-YAm29 mutant (clones 1, 3 & 5) or the pSG5 control vector (clone 1), all of which contained the *Ccnb1*::*Luc*-*MS2* transgene. We confirmed the overexpression of NF-YAm29 mutant at mRNA level, protein level and at single cell level (Supplementary Fig. S12). By RT-qPCR, we observed around 10-fold reduction in *Luciferase* mRNA and ~2–3 fold reduction in *Ccnb1* mRNA in clones expressing NF-YAm29 (Supplementary Fig. S12), consistent with earlier studies^46, 48^. We note that cell clones stably expressing NF-YAm29 had slightly lower fraction of S/G2 cells — 21–30% cells were at S/G2 while 33% of control cells (pSG5-1) were at S/G2 (Supplementary Fig. S12). However, the slight decrease (3–12%) of S/G2 cells in NF-YAm29 expressing cell clones could not explain the drastic reduction of *Luciferase* mRNA and *Ccnb1* mRNA levels.

**Figure 6:**
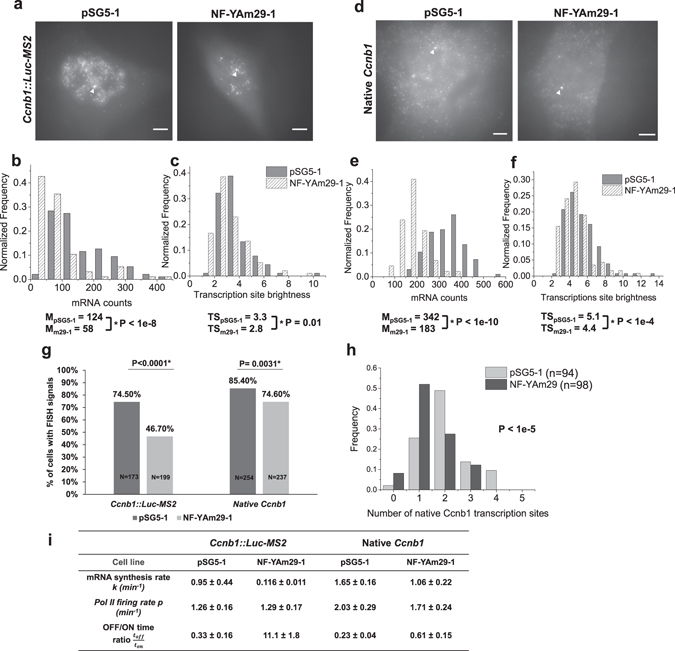
Effects of an NF-YA dominant negative mutant on *Ccnb1*::*Luc*-*MS2* transgene expression and native *Ccnb1* gene expression. (**a**) Representative single molecule RNA FISH images of the *Ccnb1*::*Luc*-*MS2* transgene using MS2 probes in control pSG5-1 cells (stably transfected with the pSG5 vector) and in cells expressing the NF-YAm29 mutant. (**b**,**c**) Histograms and medians of mRNA counts (**b**) and TS brightness (**c**) of the *Ccnb1*::*Luc*-*MS2* transgene. Measured cell number and TS number were 95 and 90 for control pSG5-1 cells, respectively, and were 96 and 96 for NF-YAm29-1 cells, respectively. (**d**) Representative single molecule RNA FISH images of the native *Ccnb1* gene in control pSG5-1 cells and in cells expressing the NF-YAm29 mutant. In panels a and d, white arrowheads indicate the transcription sites. Scale bars: 5 µm. (**e**,**f**) Histograms and medians of mRNA counts (**e**) and TS brightness (**f**) of the native *Ccnb1* gene. Measured cell number and TS number were 96 and 130 for control pSG5-1 cells, respectively, and were 88 and 116 for NF-YAm29-1 cells, respectively. In panels b,c,e and f, asterisks (*) indicate statistically significant differences (p < 0.05) between control cells (pSG5-1) and cells expressing the NF-YAm29 mutant determined by student’s t-test (P values are shown in each plot). (**g**) Fractions of cells with *Luc*-*MS2* mRNA FISH signals and native *Ccnb1* mRNA FISH signals among control cells and cells expressing the NF-YAm29 mutant. Asterisks (*) indicate statistically significant differences (p < 0.05) between the experimental groups determined by Fisher’s exact test. In panels (a–g), FISH images from one experiment using *Ccnb1* exon probes were analysed. (**h**) Frequencies of detected active TSs of the native *Ccnb1* gene using *Ccnb1* intron FISH probes among control cells and cells expressing the NF-YAm29 mutant (see images in Supplementary Fig. S17). Images were taken from two FISH experiments. Student’s t-test was used to determine the statistical significance (P value is shown in the plot). (**i**) Table summarizing calculated mRNA synthesis rates, Pol II firing rates and “OFF”/“ON” time ratios of the *Ccnb1*::*Luc*-*MS2* transgene and the native *Ccnb1* gene in control cells and cells expressing the NF-YAm29 mutant.

Next, we performed single molecule RNA FISH for *Luc*-*MS2* and native *Ccnb1* mRNA in pSG5-1 and NF-YAm29-1 cells (Fig. 6a,d). Consistent with our RT-qPCR data, we observed reduced mRNA counts for both the *Ccnb1*::*Luc*-*MS2* transgene and the native *Ccnb1* gene in cells expressing NF-YAm29 than control cells (Fig. 6b,e). Importantly, we observed similar decrease in *Ccnb1*::*Luc*-*MS2* transgene mRNA counts when we used RNA FISH probes against *Luciferase* coding region (Supplementary Fig. S13) and when we analysed other cell clones expressing NF-YAm29 using MS2 FISH probes (Supplementary Fig. S14). We also found that cells displayed uniformly- or nuclear-localized *Luc*-*MS2* mRNA and different *Ccnb1* promoter activities in the control cell line and in the pSG5-NF-YAm29 cell lines (Supplementary Fig. S15).

Because the steady state mRNA count is a ratio of mRNA synthesis rate to mRNA decay rate, reduced *Luc*-*MS2* mRNA counts in NF-YAm29 expressing cells could result from decreased mRNA synthesis rate or increased mRNA decay rate. To distinguish these possibilities, we measured steady-state levels of *Luciferase* mRNA after inhibiting transcription with 5,6-Dichloro-1-β-D-ribofuranosylbenzimidazole (DRB) and estimated mRNA half-lives. Interestingly, *Luciferase* mRNA half-lives were higher in NF-YAm29 clone-1 cells (5.8 hours) than in control cells (1.5 hours) and *Luciferase* mRNA decay rate was decreased in NF-YAm29 clone-1 cells (Supplementary Fig. S16). We thus concluded that decreased *Luc*-*MS2* mRNA counts (~2-fold) in the presence of NF-YAm29 could not result from the observed lower mRNA decay rate but instead reflected reduced *Luc*-*MS2* mRNA synthesis rate driven by the *Ccnb1* promoter.

We then calculated mRNA synthesis rates *k* and $\tfrac{{t}_{off}}{{t}_{on}}$ ratios in pSG5-1 cells and NF-YAm29-1 cells (Fig. 6i, Methods). Notably, we found over 30-fold increase in the $\tfrac{{t}_{off}}{{t}_{on}}$ ratio upon NF-YAm29 expression (increased from 0.3 to 11.1), confirming that expressing a dominant negative mutant of NF-YA substantially increased the duration of the *Ccnb1* promoter at its OFF state. Interestingly, a recent study reported that $\tfrac{{t}_{off}}{{t}_{on}}$ ratio of the *c*-*fos* gene decreased from 40 before induction to 2.5 upon transfecting multiple synthetic transcription activators binding to the *c*-*fos* promoter^23^. Live cell imaging showed that $\tfrac{{t}_{off}}{{t}_{on}}$ ratios of an integrated reporter gene decreased from 4.5 to 0.9 upon increasing Ponasterone A inducer concentrations^49^. Other studies revealed that $\tfrac{{t}_{off}}{{t}_{on}}$ ratios of the *Ctgf* gene varied from 1.2 to 7.5 upon TGF-β or serum stimulation^50^ and $\tfrac{{t}_{off}}{{t}_{on}}$ ratios of the *Oct4* and *Nanog* genes were higher in G2 than in G1^51^. Nonetheless, endogenous transcription factors regulating changes in $\tfrac{{t}_{off}}{{t}_{on}}$ ratios of these genes were not identified. Our analysis thus revealed for the first time that overexpressing a dominant-negative mutant of NF-YA substantially increased $\tfrac{{t}_{off}}{{t}_{on}}$ ratios of the *Ccnb1* promoter presumably through reducing promoter occupancies of the functional trimeric transcription factor NF-Y. As the *Ccnb1* gene is a constitutively active gene, it is interesting to note that the $\tfrac{{t}_{off}}{{t}_{on}}$ ratio of the *Ccnb1* gene was lower than those determined in several previous studies using highly inducible genes^23, 49, 50^. Furthermore, we observed that the fraction of cells expressing the *Ccnb1*::*Luc*-*MS2* transgene decreased from 75% in control cells to 40–47% in cells expressing NF-YAm29 (Fig. 6g, Supplementary Fig. S14). This finding was consistent with the notion that the OFF-time of the *Ccnb1* promoter became much longer in cells expressing NF-YAm29 so that cells without *Luc*-*MS2* mRNA (presumably decayed out before new mRNA synthesis occurred) were identified more frequently.

We note the limitations of inferring transcription kinetics based on single molecule RNA FISH data. 1) Burst ON-times and OFF-times were not directly determined. Instead, we calculated the $\tfrac{{t}_{off}}{{t}_{on}}$ ratio and mRNA synthesis rate *k* from single molecule FISH data and mRNA decay assay. 2) Equation 2 (see Methods) assumed that burst ON-time *t*_*on*_ was longer than Pol II dwell time *T*, which might not hold true for longer genes (i.e., >100 kb) but was likely valid for *Ccnb1*::*Luc*-*MS2* and the native *Ccnb1* gene in this study. 3) Pol II dwell time *T* was not measured. Instead, we assumed that Pol II dwell times were the same in control cells and in NF-YAm29-expressing cells.

Finally, we examined native *Ccnb1* gene expression upon NF-YAm29 expression by single molecule RNA FISH. Median native *Ccnb1* mRNA counts decreased from 342 in pSG5-1 cells to 183 in NF-YAm29-1 cells (Fig. 6e). Half-lives of native *Ccnb1* mRNA and control *c*-*Myc* RNA remained approximately the same in cells expressing NF-YAm29 (Supplementary Fig. S16). Thus, we calculated that mRNA synthesis rate *k* of the native *Ccnb1* gene decreased about 35% from 1.65 ± 0.16 min^−1^ to 1.06 ± 0.22 min^−1^ (Fig. 6i). We found the median TS brightness of the native *Ccnb1* gene in pSG5-1 cells and NF-YAm29-1 cells to be 5.1 and 4.4, respectively (Fig. 6f), which were equivalent to 14.2 and 12 nascent transcripts, respectively. Given the estimate that Pol II dwell time at the native *Ccnb1* gene is 7.0 ± 1.0 minutes (Fig. 3f) and the assumption that burst ON-time is longer than Pol II dwell time, we calculated that Pol II firing rate *p* was lower in NF-YAm29 expressing cells (1.71 ± 0.24 min^−1^) than in control cells (2.03 ± 0.29 min^−1^) and that the $\tfrac{{t}_{off}}{{t}_{on}}$ ratio increased from 0.23 ± 0.04 to 0.61 ± 0.15 in NF-YAm29 expressing cells (Fig. 6i).

Consistent with the observed changes in $\tfrac{{t}_{off}}{{t}_{on}}$ ratios, the fraction of cells expressing native *Ccnb1* mRNA slightly decreased from 85% in control cells to 75% in cells expressing NF-YAm29 (Fig. 6g, Supplementary Fig. S17). Furthermore, we validated these observations by performing RNA FISH using *Ccnb1* intron probes (Supplementary Table S3) and finding a significant decrease in the number of active *Ccnb1* alleles in the presence of NF-YAm29 (Fig. 6h, Supplementary Fig. S17), which indicated reduced transcription burst frequency^23^. Therefore, reduced synthesis rates of native *Ccnb1* mRNA (~35%) in the presence of NF-YAm29 likely resulted from both decreased Pol II firing rate *p* (~15%) and reduced transcription burst frequency.

### Histone modification levels at single-copy promoter transgenes

**Figure 7:**
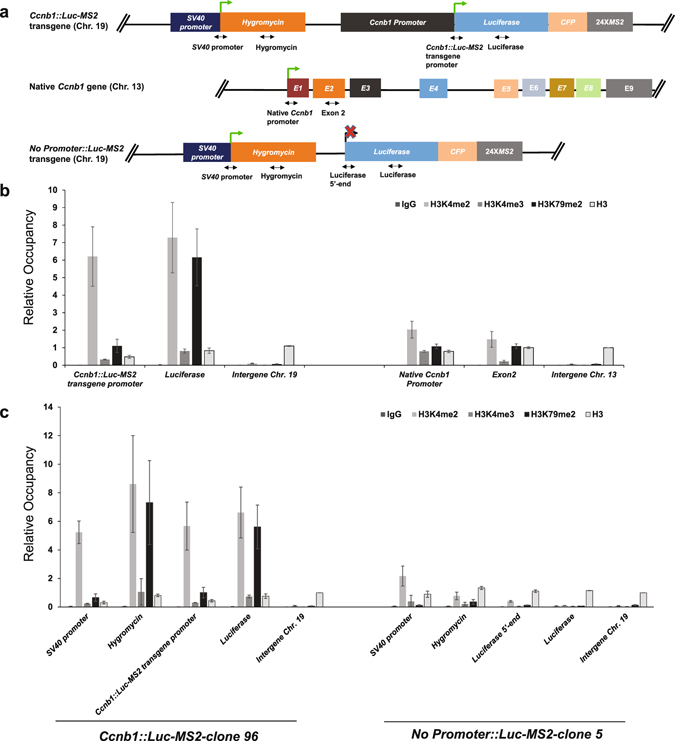
Differential enrichment of active histone modifications and nucleosome occupancy at the *Ccnb1*::*Luc*-*MS2* transgene locus, the native *Ccnb1* gene and the *No Promoter*::*Luc*-*MS2* transgene locus. (**a**) Schematic of the *Ccnb1*::*Luc*-*MS2* transgene locus, the native *Ccnb1* gene, the *No Promoter*::*Luc*-*MS2* transgene and the locations of ChIP-qPCR amplicons at each gene locus. Distances between *Ccnb1*::*Luc*-*MS2* transgene promoter and *Luciferase* amplicons and between native *Ccnb1* promoter and exon 2 amplicons are 686 bp and 1,238 bp, respectively. The Chr. 19 intergene amplicon is located at 7.5 kb upstream of the FRT insertion site. The Chr. 13 intergene amplicon is located at 10.4 kb upstream of 5′-end of the native *Ccnb1* gene. (**b**) ChIP analysis for H3K4me2, H3K4me3, H3K79me2 and histone H3 at the *Ccnb1*::*Luc*-*MS2* transgene and the native *Ccnb1* gene in *Ccnb1*::*Luc*-*MS2*-clone 96 cells. (**c**) ChIP analysis for H3K4me2, H3K4me3, H3K79me2 and histone H3 at the *Ccnb1*::*Luc*-*MS2* transgene from *Ccnb1*::*Luc*-*MS2*-clone 96 cells, and at the *No Promoter*::*Luc*-*MS2* transgene from *No Promoter*::*Luc*-*MS2*-clone 5 cells, respectively. In panels b and c, relative occupancies were determined by the percent input of each ChIP DNA by antibodies against each histone mark normalized to percent input of histone H3 enrichment at the *Ccnb1* intergenic region on chromosome 13. Error bars represent standard deviations of three independent experiments, each performed in duplicate.

Our single-copy promoter transgene integrated at an identified genetic locus allows carrying out molecular analysis, such as comparing histone modification levels at the promoter transgene versus at the native gene by ChIP while quantifying their expression by single mRNA counting. We designed gene-specific primers to compare histone modification levels at the *Ccnb1*::*Luc*-*MS2* transgene and at the native *Ccnb1* gene (Fig. 7a, Supplementary Table S4), both of which had similar Pol II densities as detected by single molecule RNA FISH (Figs 2j and 3f). We made several interesting observations. First, H3K4me2 levels at both promoter and gene body and H3K79me2 level at gene body of the *Ccnb1*::*Luc*-*MS2* transgene were much higher than those of the native *Ccnb1* gene (Fig. 7b). Second, H3K4me3 level was higher at promoter than at gene body of the native *Ccnb1* gene, while H3K4me3 level was higher at gene body than at promoter of the *Ccnb1*::*Luc*-*MS2* transgene (Fig. 7b). This could be due to the first exon of the *Ccnb1*::*Luc*-*MS2* transgene being much longer than the native *Ccnb1* gene, as the first exon length was shown to correlate with H3K4me3 levels at promoters^52^. Third, we observed lower histone H3 occupancy at the *Ccnb1*::*Luc*-*MS2* transgene promoter than at the native *Ccnb1* promoter (Fig. 7b), suggesting a more open chromatin structure at the *Ccnb1*::*Luc*-*MS2* transgene promoter than the native *Ccnb1* promoter.

Because the transgene locus contains an active *Hygromycin* resistance gene driven by the *SV40* promoter (Fig. 7a) that may affect histone modification levels, we compared histone modification profiles of *Ccnb1*::*Luc*-*MS2* transgene with the *No promoter*::*Luc*-*MS2* transgene. We found near baseline levels of active histone marks at the *No promoter*::*Luc*-*MS2* transgene (Fig. 7c), indicating that the active *Hygromycin* gene driven by the *SV40* promoter does not spread active histone marks across the entire locus. However, H3K4me2 and H3K79me2 levels at the *Hygromycin* gene were higher at cells containing the *Ccnb1*::*Luc*-*MS2* transgene than at cells containing the *No promoter*::*Luc*-*MS2* transgene (Fig. 7c), suggesting that the neighboring active *Ccnb1* promoter and active *SV40* promoter may mutually enhance H3K4me2 and H3K79me2 levels at each other, but not H3K4me3 levels. Furthermore, although higher H3K4me2 and H3K79me2 levels and reduced H3 occupancy are correlated with higher gene expression in general, our analysis showed that these chromatin features at the *Ccnb1*::*Luc*-*MS2* transgene do not predispose the *Ccnb1* promoter to higher transcription activities than the native *Ccnb1* gene.

## Discussion

In this paper, we have developed and validated an experimental approach that allows studying promoter regulation *in vivo* by site-specific integration of single-copy promoter transgenes in mouse cells. Using this approach, different promoter transgenes can be integrated at an identified genetic locus and their expression can be compared with each other and with the endogenous genes. Our work showed that the *Ccnb1* promoter when integrated at this locus, could drive transgene expression to comparable levels of the native *Ccnb1* gene. A recent study also developed a promoter isolation assay that showed several yeast promoters could recapitulate the expression of endogenous genes when inserted upstream of the *MDN1* gene^53^. Our study thus provides a novel experimental system to study regulation of promoter activation at the single cell and single mRNA level in mammalian cells. In addition to *Ccnb1*, other housekeeping gene promoters, inducible promoters or developmentally regulated promoters such as muscle-specific promoters may also be studied accordingly. Live cell imaging^15, 17^ can be used to directly measure the key regulated kinetic steps during transcription activation (such as initiation rates, elongation rates, burst ON-times and OFF-times).

Our study revealed a modest increase of *Ccnb1* gene expression and *Ccnb1*::*Luc*-*MS2* transgene expression (both in mRNA counts and nascent transcript counts) in G2/M compared to G1. This result closely resembles the result from the nuclear run-off assay of the human *Ccnb1* gene in Hela cells^27^, and supports that the chromosomally-integrated *Ccnb1* promoter recapitulated the cell cycle regulated changes in native *Ccnb1* gene expression. Changes in expression during the cell cycle were reported for *Oct4* and *Nanog* genes^51^, i.e. increase in gene copy number from 2 to 4 in G2 was accompanied with decreased nascent transcript counts at each active allele^51^. This gene dosage compensation effect was achieved by decrease in gene activation rate *k*_*on*_^51^. In contrast, in our study we observed increased nascent transcript counts of the *Ccnb1* promoter at G2/M and thus an increase in Pol II firing rate *p*. Transcription factors regulating this increase in Pol II firing rates at the *Ccnb1* promoter in G2/M remain to be determined.

Our study revealed distinct cell populations with nuclear or uniformly-localized *Ccnb1*::*Luc*-*MS2* transgene mRNA that exhibited higher or lower mRNA counts/nascent transcript counts, respectively. Our results thus suggested that the ON/OFF state used to describe transcription bursting of mammalian promoters is not a simple telegraph model (at least for the *Ccnb1* promoter). Instead, the ON state may constitute multiple promoter activation states during which Pol II initiates from the *Ccnb1* promoter at different firing rates. Previous studies in mouse ES cells identified two production rates of the *Nanog* promoter^54^ and the intrinsic noise of *Nanog* gene expression among two active alleles^55^. However, these work did not show whether cell-to-cell variations of *Nanog* gene expression were cell cycle-independent, while our study revealed that individual G1 cells or G2/M cells exhibited variable *Ccnb1* promoter activation states. In future studies, the molecular basis underlying cell-to-cell variations of *Ccnb1* promoter activation should be defined. It is also likely that the *Ccnb1* promoter is not unique in exhibiting variable promoter activation states at the single cell level. Our experimental system allows generating different promoter transgenes at the same genetic locus and can be used to study additional promoters or promoter mutants for comparison. Furthermore, studies in yeast showed that promoter sequences can influence mRNA localization and stability^56, 57^. Whether transcriptional activation of the *Ccnb1* promoter or other promoters is coupled to mRNA nuclear export and/or localization in mammalian cells is an interesting topic for future studies.

How endogenous transcription factors regulate transcription kinetics has largely escaped analysis. Two previous reports studied the effects of synthetic transcription activators or serum response factor on *c*-*Fos* and *β*-*actin* gene expression, respectively^23, 58^. In our work, we found that overexpressing a dominant-negative mutant of NF-YA (NF-YAm29) reduced expression of both the *Ccnb1*::*Luc*-*MS2* transgene and the native *Ccnb1* gene by increasing $\tfrac{{t}_{off}}{{t}_{on}}$ ratios. Thus, we confirmed that a major function of the sequence-specific transcription factor NF-Y is to regulate the kinetics of the *Ccnb1* promoter turning “on” and “off”. Interestingly, we also observed differential effects on transcription kinetics of the *Ccnb1*::*Luc*-*MS2* transgene and the native *Ccnb1* gene by NF-YAm29 expression. 1) Transgene expression was reduced to a larger degree than the native *Ccnb1* gene. 2) Upon NF-YAm29 expression, the $\tfrac{{t}_{off}}{{t}_{on}}$ ratio of the *Ccnb1*::*Luc*-*MS2* transgene was increased to a much larger degree than that of the native *Ccnb1* gene (Fig. 6i), suggesting that additional transcription activators may function in maintaining the ON state of the native *Ccnb1* gene upon NF-Y loss-of-function. By examining Hi-C data^59^, we didn’t find genomic regions interacting with the native *Ccnb1* promoter that may act as distal enhancers. 3) We found no changes in Pol II firing rate for the *Ccnb1*::*Luc*-*MS2* transgene but reduced Pol II firing rate for the native *Ccnb1* gene upon NF-YAm29 expression (Fig. 6i). Thus, roles of NF-Y in Pol II recruitment may slightly differ between the *Ccnb1*::*Luc*-*MS2* transgene and the native *Ccnb1* gene. Future studies can be performed to study the roles of NF-YB/YC and key transcription coactivators in regulating transcription kinetics of the *Ccnb1* promoter. For example, depleting transcription coactivators that recruit Pol II to the *Ccnb1* promoter may result in reduced Pol II firing rates during the ON state, while depleting factors that assemble transcription preinitiation complexes may result in the increased OFF-time.

Finally, the single-copy transgene approach enables coupling transcription imaging with molecular analysis. In this study, we compared histone modifications levels of the native *Ccnb1* gene and the *Ccnb1*::*Luc*-*MS2* transgene by ChIP and measured their expression at the absolute level by single molecule RNA FISH in the same cell population. Although active histone modifications are generally correlated with gene expression^60, 61^, we provided some new insights in this study. We found that the two neighboring active promoters (*SV40* and *Ccnb1*) on the transgene locus can synergistically enhance H3K4me2 and H3K79me2 levels on each other. However, higher levels of H3K4me2 and H3K79me2 at the *Ccnb1*::*Luc*-*MS2* transgene did not result in a higher level of expression than the native *Ccnb1* gene. Thus, H3K4me2 and H3K79me2 may function in stabilizing the active chromatin environment permissive for transcription, but the elevated levels of these two active histone marks do not predispose higher transcription rates of the *Ccnb1* promoter. This experimental system therefore provides an opportunity to site-specifically modulate the level of individual active histone modifications and to understand their functions in regulating transcription kinetics.

## Methods

### DNA constructs

The reporter vector containing the *Ccnb1*::*Luc*-*MS2* promoter transgene was generated as the following. The *CMV* promoter from the *pcDNA5/FRT* vector (Invitrogen) was removed and replaced with a 2.78 kb mouse *Ccnb1* promoter. The *Luciferase-CFP-24XMS2* cassette was digested from the *pGL3-MS2* vector (a gift from the Tjian lab) and inserted into the multiple cloning site of the modified *pcDNA5/FRT* reporter vector downstream of the *Ccnb1* promoter. The reporter vector containing the No-promoter transgene was generated by removing the *Ccnb1* promoter sequence from the transgene reporter vector through digesting with restriction enzymes, treating with Klenow DNA polymerase (New England Biolabs) and re-ligating the vector. All DNA constructs were verified by sequencing.

### Cell culture

C2C12 mouse myoblasts were obtained from American Type Culture Collection (ATCC) and were cultured in Dulbecco’s Modified Eagle Medium (DMEM, high glucose, Invitrogen) supplemented with 10% fetal bovine serum (Sigma) and 1X penicillin/streptomycin (Invitrogen). To obtain the majority of C2C12 myoblasts arrested at G1 or G2/M, cells were treated with 0.4 mM mimosine for 24 hours and 40 ng/ml nocodazole for 16 hours, respectively. To induce myocyte differentiation, C2C12 myoblasts were grown to 100% confluency and were switched to the differentiation media (DMEM + 2% horse serum) for 3 days with daily media change. To make stable cell lines with the pSG5 vector or the pSG5-NF-YAm29 vector, cells were transfected with plasmids using Lipofectamine 2000 (Invitrogen) according to the manufacturer’s protocol.

### Generation of C2C12 Flp-In cell lines and characterization of the integration locus of the FRT site

C2C12 Flp-In cell lines were generated following the instructions by Invitrogen. Briefly, the pFRT/LacZeo plasmid (Invitrogen) was linearized by ScaI and was transfected into proliferating C2C12 myoblasts cultured in 6-well plates in limiting amounts (0.1 to 0.5 µg/well). Cells were then switched to selection media (Growth media with 500 µg/ml Zeocin (Invitrogen)) for two weeks with media change every three to four days. After 10–14 days, cell foci were picked by 8-mm diameter cloning cylinders (Fisher Scientific) into 24-well plates and individual cell clones were grown and expanded into 10-cm plates in the growth media with 250 µg/ml Zeocin. Genomic DNA was isolated from each cell clone by DNeasy kit (Qiagen). Splinkerette PCR was performed to amplify the genomic sequences adjacent to the pFRT/LacZeo plasmid sequence, following a similar protocol for mapping transposon insertion sites in the *Drosophila* genome^62^. Among the majority of cell clones that contain multiple copy insertions of the pFRT/LacZeo plasmid, splinkerette PCR products only contained concatenate plasmid sequences spanning the linearization site which had an identical size and were verified by sequencing (Supplementary Fig. S1). In a small fraction of cell clones containing single-copy insertions, splinkerette PCR products showed distinct sizes (Supplementary Fig. S1) and contained the plasmid sequence ligated to the genomic DNA sequence of the insertion site. We obtained several cell clones in which splinkerette PCR products were sequenced to identify insertion sites mapped to mouse chromosome 4, 7 and 19, respectively. We chose the 5A5 Flp-In cell clone mapped to chromosome 19 (~53 Mb, Supplementary Fig. S1) for further work, because promoter transgenes integrated into this locus were actively transcribed.

### Integration of promoter transgenes

Promoter transgenes were integrated into the 5A5 Flp-In cell clone by co-transfecting the reporter constructs with the pOG44 plasmid (Invitrogen) encoding a modified yeast Flp enzyme with optimal stability at 37 °C and reduced recombinase activity. We determined that 9:1 mass ratio of pOG44 to the transgene reporter vector gave optimal number of cell clones. The pcDNA5/CAT/FRT plasmid (Invitrogen) was co-transfected with pOG44 into the 5A5 cell clone as a positive control. Transfection was performed in six-well plates. Twenty-four hours after transfection, cell culture media was replaced with growth media without antibiotics. Forty-eight hours after transfection, transfected cells from each well were trypsinized and resuspended in the selection media (growth media +1 mg/ml Hygromycin). We changed the selection media every three or four days. Cell foci became visible usually 8–14 days after plating. We picked individual cell clones by 8-mm diameter cloning cylinder (Fisher Scientific) and resuspended them into 24-well plates. Cell clones were expanded into 10-cm plates. We then froze cells and isolated genomic DNA (DNeasy kit, Qiagen) and total RNA (RNeasy Plus kit, Qiagen) for analysis.

### Screening cell clones containing promoter transgenes

We performed the following steps to screen cell clones. First, we performed genomic DNA PCR to identify cell clones with correct integration of transgene reporter vectors and sequenced the PCR products to confirm the correct integration. Second, we performed reverse transcription followed by quantitative PCR (RT-qPCR) to measure the expression of *Luciferase* mRNA relative to *Gapdh* mRNA in each cell clone. Cell clones with correct integration and *Luciferase* mRNA expression were subject to further analysis. We note that the loss of *LacZ*-*Zeocin* expression (by β-Gal assay or gain of Zeocin sensitivity) did not always occur in cell clones with verified integration and *Luciferase* mRNA expression (Supplementary Fig. S2). We identified *Ccnb1*::*Luc*-*MS2* clone 96 and clone 134 for further analysis.

### Single molecule RNA FISH

Single molecule RNA FISH was performed as described^15^. Briefly, three 20-mer oligonucleotides targeting the inter-stem loop region of the 24X MS2 repeats^15^ were labeled with amino groups at both 5′- and 3′-ends. These oligonucleotides were incubated with 5′(6′)-carboxytetramethylrhodamine N-succinimidyl ester (Invitrogen) at room temperature overnight. Labeled oligo probes were purified by ethanol precipitation. Stellaris FISH probes for *Luciferase* mRNA, native *Ccnb1* mRNA and *Ccnb1* intron regions were designed and purchased from Biosearch Technologies. For FISH, cells were grown on two-well Lab-Tek CC2 chamber slides (ThermoFisher Scientific), washed once with 1X PBS and fixed with 4% paraformaldehyde for 15 minutes. Cells were then permeabilized with 1X PBS + 0.05% Triton X-100 for 10 min, washed once with PBSM (1X PBS + 5 mM MgCl_2_), incubated with the prehybridization buffer (10% formamide, 2X SSC, 2 mg/ml BSA, 0.2 mg/ml yeast tRNA, 0.2 mg/ml sheared salmon sperm DNA and 10% dextran sulfate) at 37 °C for 10 minutes. 10–20 ng of labeled oligo probes were mixed with the prehybridization buffer and added to the slide. Cells were hybridized with probes at 37 °C overnight. Cells were washed twice for 20 minutes with the prehybridization solution at 37 °C, 10 minutes in 2X SSC at room temperature and 10 minutes in PBSM at room temperature. Cells were then counterstained with Hoechst33342 (0.5 µg/ml) in 1X PBS before mounting in VectaShield (Vector Labs).

### Microscope imaging and single molecule RNA FISH analysis

A custom-built microscope based on an Olympus IX-81 inverted microscope was used to acquire single molecule RNA FISH images. A 60X water-immersion objective (Olympus UPlanApo, numerical aperture 1.2) was used to acquire high-resolution FISH images of individual cells. A 20X air objective (Oympus UPlanApo, numerical aperture 0.75) was used to acquire images of multiple cells for counting the fraction of cells with mRNA expression. A 2X magnification changer was used at all times during image acquisition. A 561 nm laser was used to illuminate the sample at ~5 mW for MS2 FISH or ~20 mW for *Luciferase* and *Ccnb1* mRNA FISH. Fluorescence was collected by a dichroic mirror (FF568-Di01-25X36, Semrock, Inc.) and an emission filter (FF01-617/73-25, Semrock Inc.). A charge-coupled device camera (Hamamatsu C9100-13) was used to acquire the FISH images at the non-electron multiplying mode. The Metamorph software (Molecular Devices) was used to acquire images. Z-stacks of 40 images were taken for each cell at 0.2 µm per step with 800 msec exposure time. Images were analysed by the AirLocalize software (kindly provided by Drs Timothee Lionnet and Robert Singer). Counts of diffraction-limited fluorescence spots per cell gave the value of total mRNA counts. To measure TS brightness, fluorescence intensity of the TS was normalized by the average fluorescence intensity of an mRNA.

### Calculating “OFF”/“ON” time ratios

Transcription of most mammalian mRNA genes occurs in bursts in which genes alternate between ON and OFF states^3, 4, 21, 23^. At ON state, RNA Pol II initiates transcription from promoters and synthesizes mRNA. During one ON period, multiple RNA Pol II can initiate from promoters and some of them complete productive elongation to produce pre-mRNA. If we assume that the rate of firing productively-transcribing Pol II in an ON period (i.e., Pol II firing rate) is *p*, the average time of ON and OFF periods are *t*_*on*_ and *t*_*off*_, respectively, mRNA synthesis rate *k* can be shown as:

1 $$k=\frac{p}{1+\tfrac{{t}_{off}}{{t}_{on}}}$$

Modulation of mRNA synthesis rate *k* can be described by altering Pol II firing rate *p* or altering the $\tfrac{{t}_{off}}{{t}_{on}}$ ratio. The former parameter is related to the regulation of transcription initiation and early elongation, while the latter parameter is related to the regulation of transcription burst kinetics. Because Pol II firing rate *p* is the ratio of nascent transcript counts *c* to Pol II dwell time *T*,

2 $$p=\tfrac{c}{T}$$

The “OFF”/“ON” time ratio can be shown as:

3 $$\tfrac{{t}_{off}}{{t}_{on}}=\frac{c}{T\cdot k}-1$$

### Chromatin immunoprecipitation (ChIP)

ChIP protocol was described in the Supplementary Methods section. The following antibodies were used: rabbit anti-H3 (Abcam, ab1791), rabbit anti-H3K4me3 (Abcam, ab8580), rabbit anti-H3K4me2 (Abcam, ab7766) and rabbit anti-H3K79me2 (Abcam, ab3594). qPCR was performed with a Bio-Rad CFX96 Touch Real-time PCR Detection system using the SYBR Green qPCR Master Mix (Bio-Rad).

### Statistical analysis

Single molecule RNA FISH data were analysed using the Origin 9.1 program (OriginLab Corporation). Comparisons between experimental groups were performed using an unpaired Student’s *t*-test or Fisher’s exact test. In all cases, P values < 0.05 were considered statistically significant.

## Electronic supplementary material

Supplementary Information
