# Supplementary material for: Single-cell, single-mRNA analysis of Ccnb1 promoter regulation
Source: Sci Rep. 2017 May 18;7:2065. doi: 10.1038/s41598-017-02240-y (PMC5437063; doi:10.1038/s41598-017-02240-y)
Supplement: Supplementary file 1 — Supplementary Information [file 41598_2017_2240_MOESM1_ESM.pdf]

Supplementary Information

Single-cell, single-mRNA analysis of *Ccnb1* promoter regulation

Nidhi Vishnoi[1] and Jie Yao[1, *]

[1] Department of Cell Biology, Yale University School of Medicine, New Haven, CT 06510, USA

[*] To whom correspondence should be addressed. Tel: 1-203-737-6897; Fax: 1-203-785-7446;

Email: yao.j@yale.edu

Supplementary Results

Expression of the *Ccnb1::Luc-MS2* transgene mimics the native *Ccnb1* gene in synchronized cells

To reproduce and validate our findings on *Ccnb1* promoter regulation during the cell cycle, we performed cell cycle synchronization experiments to examine cells that were blocked at G1 and progressed into G2 (6 hours after release) and into the next G1 phase (10 hours after release) (Supplementary Fig. S7). We then examined *Ccnb1::Luc-MS2* transgene and native *Ccnb1* expression by single molecule RNA FISH (Supplementary Fig. S7). As expected, G2 cells had higher mRNA counts for both the *Ccnb1::Luc-MS2* transgene and the native *Ccnb1* gene than G1 cells (Supplementary Fig. S7). We note that mRNA counts in synchronized G2/M or G1 cells were lower than mRNA counts in G2/M-arrested or G1-arrested cells (Fig. 4b, e), likely due to the prolonged cell cycle arrest induced by nocodazole/mimosine that may increase cell volumes resulting in higher mRNA counts [1,2]. Importantly, we observed reduced transcription site (TS) brightness at both the *Ccnb1::Luc-MS2* transgene and the native *Ccnb1* gene in G1 than in G2/M (Supplementary Fig. S7). Under the same assumptions used in this study, we estimated that the average Pol II density at the native *Ccnb1* gene was 1.42 kb^{-1} and 1.74 kb^{-1} in G1 and G2/M-synchronized cells, respectively and that the average Pol II firing rate at the native *Ccnb1* gene was 1.57 ± 0.22 min^{-1} and 1.93 ± 0.28 min^{-1} in G1 and G2/M-synchronized cells, respectively. We observed that a higher fraction of cells expressed both the *Ccnb1::Luc-MS2* transgene and the native *Ccnb1* gene in G2/M than in G1 (Supplementary Fig. S7). Therefore, we confirmed that expression of the *Ccnb1* promoter transgene mimics the native *Ccnb1* gene during the cell cycle. Additionally, about 40% of G2/M-synchronized cells had two

active *Ccnb1* alleles while only ~20% of G1-synchronized cells contained two active *Ccnb1* alleles (Supplementary Fig. S7).

Distinct subcellular localizations of *Luc-MS2* mRNA correlate with *Ccnb1* promoter activities among G1 or G2/M cells.

One of the explanations for the observed cell subpopulations with distinct *Luc-MS2* mRNA localizations among asynchronous cells (Fig. 5a) is that the subpopulation of cells with nuclear-enriched *Luc-MS2* mRNA are in G2/M phase when the *Ccnb1* promoter has a higher activity. To test this, we first examined FISH data of cells arrested at G1 and G2/M by mimosine and nocodazole treatment. We identified that 36% cells and 47% cells exhibited nuclear-enriched *Luc-MS2* mRNA after mimosine treatment (G1) and nocodazole treatment (G2/M), respectively (Supplementary Fig. S10). As found in asynchronous cells (Fig. 5), cells with nuclear-enriched *Luc-MS2* mRNA display higher mRNA counts and higher TS brightness than cells with uniformly-localized *Luc-MS2* mRNA in mimosine and nocodazole-treated cells (Supplementary Fig. S10), confirming that higher *Ccnb1* promoter activity correlates with mRNA nuclear retention among cells blocked at G1 or G2/M and suggesting that variable *Ccnb1* promoter activation states observed at individual cell level are cell cycle-independent.

Furthermore, we examined FISH images of cells synchronized in G2 and the next G1 phase. We confirmed the existence of two cell populations that differed in *Ccnb1* promoter activity and *Luc-MS2* mRNA localization among synchronized G1 or G2/M cells (Supplementary Fig. S11). We identified that 39% cells and 69% cells exhibited nuclear-enriched transgene RNA in synchronized G1 or G2/M cells, respectively (Supplementary Fig. S11). Cells with nuclear-enriched *Luc-MS2* mRNA generally had higher levels of mRNA counts

and TS brightness than cells with uniformly-localized *Luc-MS2* mRNA (Supplementary Fig. S11). Taken together, our study identified cell-to-cell variations in *Ccnb1* promoter activities and *Luc-MS2* mRNA localizations among asynchronous cells, G1- or G2/M-arrested cells and among cells synchronized at G1 or G2/M. Therefore, variable activation states of the *Ccnb1* promoter likely exist among individual cells during each cell cycle stage.

Differential changes in *Luciferase* and *Ccnb1* expression detected by qPCR and by FISH during the cell cycle

We noticed some differences in *Ccnb1::Luc-MS2* transgene expression and native *Ccnb1* gene expression detected by FISH or by RT-qPCR. Compared to asynchronous cells, *Luciferase* mRNA levels decreased more than 2-fold in G1 and increased by ~50% in G2 as detected by RT-qPCR, resulting in an approximately ~4-fold increase of *Luciferase* mRNA in G2 than in G1 (Supplementary Fig. S5). In contrast, we observed by FISH that the median *Luc-MS2* mRNA counts were 302, 335 and 426 in asynchronous, G1 and G2 cells, respectively (Figs. 2c, 4b). Thus, *Luc-MS2* mRNA counts only increased by 30-50% in G2 cells compared to asynchronous cells or G1 cells. Likewise, native *Ccnb1* mRNA levels decreased by ~25% in G1 and increased by ~2-fold in G2 (compared to asynchronous cells), resulting in an ~2.5-fold increase of *Ccnb1* mRNA in G2 than in G1 (Supplementary Fig. S5). In contrast, FISH detected about ~50% higher native *Ccnb1* mRNA counts in G2 cells than asynchronous cells or G1 cells (Figs. 3c, 4e, median mRNA counts = 391, 406 and 593 in asynchronous, G1 and G2, respectively). Therefore, both RT-qPCR and FISH observed increased *Luciferase* and *Ccnb1* mRNA levels in G2 cells than in asynchronous cells or G1 cells. However, the two methods differed in that decreased *Luciferase* and *Ccnb1* mRNA levels in G1-arrested cells than in asynchronous cells were observed by RT-

qPCR (Supplementary Fig. S5) but were not detected by FISH (Figs. 2c, 3c, 4b, 4e). Because approximately two-thirds of asynchronous cells were in G1 (Supplementary Figs. S5, S12), a 2-3 fold decrease of *Luciferase* mRNA in G1 cells than asynchronous cells would require a 4-8 fold higher *Luciferase* mRNA in S/G2 cells than in G1 cells among the asynchronous population. Although MS2 RNA FISH demonstrated excellent dynamic range in detecting *Luc-MS2* transgene expression (Figs. 2a, d), we did not observe such a 4-8 fold higher *Luc-MS2* mRNA count in G2 than in G1 either in arrested cells (Fig. 4b) or synchronized cells (Supplementary Fig. S7), nor could we distinguish two cell populations differing in mean *Luc-MS2* mRNA counts among asynchronous cells (Fig. 2c). Because RT-qPCR can be subject to artifacts during cell lysis and RNA isolation while FISH quantitation is sensitive to enlarged cell volumes [1,2], we note here that one should be cautious to interpret conflicting FISH and RT-qPCR data on gene expression changes.

Supplementary Methods

Splinkerette PCR

Splinkerette PCR was carried out as described [3]. Briefly, genomic DNA was extracted and digested with BstYI at 60 ºC. The purified digested genomic DNA was ligated to annealed Splinkerette oligonucleotide (Supplementary Table S5) and two rounds of PCR were carried out with Phusion Taq polymerase (NEB) and PCR primers (Supplementary Table S5). The final PCR products were resolved in agarose gel. The PCR products were then treated with Antarctic phosphatase (NEB) and Exonuclease I (NEB) and subjected to sequencing.

Luciferase reporter activity assays

C2C12 cells were plated in a 24-well plate at a density of 4×10^4 cells/well and transfected 24 hours later with 180 ng of pGL3 empty vector (Promega) or pGL3 vectors containing four *Ccnb1* promoter deletion mutants (Supplementary Fig. S2). Renilla vector pRL (Promega) (20 ng) was co-transfected to normalize for the transfection efficiency. Luciferase activity was measured 48 hours after transfection using the Dual Luciferase Reporter Assay System according to the manufacturer's instructions (Promega).

RNA extraction and real-time PCR analysis

Total RNA was isolated using the RNeasy Plus Mini Kit (Qiagen). Four hundred nanograms of total RNA was converted to cDNA using SuperScript III Reverse Transcriptase (Invitrogen) in conjunction with oligo(dT) primers (Invitrogen). The resulting cDNA samples were subjected to real-time PCR using gene-specific primers and iTaq Universal SYBR® Green Supermix (Bio-

Rad). Real-time PCR was performed in a CFX-96 Touch Real-Time PCR Detection System (Bio-Rad) and the data were analyzed using the CFX Manager software (Bio-Rad).

Measurement of mRNA decay

mRNA half-lives were calculated by measuring mRNA levels at distinct time points after adding the transcription inhibitor 5,6-Dichlorobenzimidazole 1-β-D-ribofuranoside (DRB) (Sigma) at a final concentration of 30 µg/ml into the culture media. Total RNA was extracted at the time points 0, 0.5 hour, 1 hour, 2 hours and 4 hours after adding DRB. RT-qPCR was carried out using *18S* rRNA to normalize the expression. mRNA decay rates were determined as previously described [4]. Briefly, $ln(2^{-(Ct_{mRNA}-Ct_{18S})})$ at distinct time points after DRB treatment were plotted on the y-axis and the corresponding time points were plotted on the x-axis. k_{decay} was determined by linear regression, and mRNA half-life $T_{\frac{1}{2}}$ was calculated as $T_{\frac{1}{2}} = \frac{Ln2}{k_{decay}}$. Three independent experiments were carried out and the average $T_{\frac{1}{2}}$ was calculated.

Propidium iodide (PI) staining and cell cycle analysis by flow cytometry

Cells were trypsinized and centrifuged at 1,200 rpm for 5 min and were washed with cold 1X PBS three times. The cell pellet was re-suspended in 400 µl 1X PBS and 1 ml cold 100% ethanol was added slowly while vortexing. The cell suspension was incubated overnight at 4°C. For PI staining, cells were pelleted, washed three times with 1X PBS and re-suspended in 1X PBS. Cells were then incubated with RNase A (final concentration 100 µg/ml) and PI (final concentration 50 µg/ml) for 30 min at room temperature (RT). The stained cells were immediately analyzed on a Stratedigm flow cytometer (FACS facility, Yale School of Medicine)

for relative DNA content. Collected data was analyzed using the FlowJo software (FlowJo, LLC).

Cell cycle synchronization

Cells were synchronized at G1 and G2/M phases according to Heintz et al [5]. Briefly, cells were plated at around 40-60% confluency in growth media containing 2 mM thymidine and incubated for 12 hours. Cells were then released from the block by three washes in fresh media minus serum, trypsinized and suspended in growth media containing 24 µM thymidine and deoxycytidine. Cells were then plated in Nunc Lab-Tek CC2 two-well chamber slides (ThermoFisher Scientific) for single molecule RNA FISH and on 10 cm plates for flow cytometry analysis. 9 hours after plating cells, the media was replaced with media containing 5 µg/ml aphidicolin for an additional 12 hours. Cells were then released from the aphidicolin block by washing with fresh media minus serum and then grown in complete media. Cells synchronized at G2/M or G1 were analyzed by FISH or flow cytometry at 6 hours or 10 hours post-release, respectively.

Combined RNA FISH and DNA FISH

RNA FISH probes against MS2 were generated by nick translation of MS2 repeats purified from AluI digestion of the pGL3-MS2 vector. Nick translation was carried out using BioProbe Nick Translation DNA labeling system (Enzo Life Sciences) in presence of Cy3-dUTP labelling Mix (0.5 mM dATP, 0.5 mM dCTP, 0.5 mM dGTP, 0.25 mM dTTP, 0.25 mM Cy3-dUTP (GE Healthcare), pH 7.5). The probes were then purified using MinElute PCR Purification Kit (Qiagen). DNA FISH probe against the transgene insertion site was generated by Atto488 NT

labeling Kit (Jena Bioscience) using bacteria artificial chromosome (BAC) clone RP23-370K1. DNA FISH was carried out in conjunction with RNA FISH according to the published protocol [6] with slight modifications. Briefly, cells were fixed with 4% paraformaldehyde for 10 min. Cells were washed once with 1X PBS, permeabilized with 0.5% Triton X-100 in 1X PBS for 10 min, followed by washing with PBST (1X PBS (DEPC treated) + 0.05% Tween-20) for three times and 2X SSC for 5 min. Cells were then denatured in 50% formamide and 2X SSC for 30 min at 80°C. Next, cells were incubated with 25 µl hybridization buffer containing 50% formamide, 2X SSC, 50 mM phosphate buffer, 10% dextran sulfate, 0.7 mg/ml sonicated salmon sperm DNA, 0.1 mg/ml mouse Cot1 DNA, 0.4 U/µl RNaseOUT and 200 ng labeled DNA FISH and RNA FISH probes (100 ng each). The hybridization mix was denatured at 80°C for 3 min. Slides were sealed with rubber cement, denatured at 80°C heat block for 3 min and incubated overnight at 37°C. Cells were then washed with 2X SSC + 50% formamide for 1 hour at 37°C, washed with 2X SSC at room temperature (RT) and then with PBST. Samples were mounted with Vectashield (Vector Laboratories) and imaged in an Olympus IX-81 inverted wide-field microscope. A 488nm laser was used to image DNA FISH signals. Green fluorescence was collected by a 525/50 Brightline bandpass filter and imaged in z-stacks (z-distance = 0.2 µm). RNA FISH signals were imaged in the red channel as described in the main text.

Combined single molecule RNA FISH and immunofluorescence staining

Single molecule RNA FISH was performed as described in the main text. To continue with immunofluorescence staining, cells were fixed again with 4% paraformaldehyde for 15 min. Cells were washed once with 1X PBS and then permeabilized with 1X PBS + 0.25% Triton X-

100 for 10 min. Cells were washed three times with PBST (1X PBS + 0.05% Tween-20) for 5 min each and blocked with 2% Ultrapure BSA (Ambion) in 1X PBS in presence of 0.4 U/µl RNaseOUT (Invitrogen) for 30 min at RT. Cells were then incubated with 1:500 goat-anti-Lamin B antibody (Santa Cruz Biotechnology, sc-6217) in 2% BSA in 1X PBS and 0.4 U/µl RNaseOUT for 1 hour at RT. Cells were washed three times in DEPC-treated 1X PBS before incubating with anti-goat Alexa Flour 488 conjugate (A-11055, ThermoFisher Scientific) at 1:1000 dilution for 1 hour at RT. Cells were then mounted in VectaShield (Vector Labs) and imaged in an Olympus IX-81 wide-field microscope (immunofluorescence in the green channel and RNA FISH in the red channel) as described above.

Western blotting

Cells were lysed in RIPA buffer (50 mM Tris, pH 7.4, 150 mM NaCl, 1% Triton X-100, 0.5% deoxycholate, 0.1% SDS) supplemented with 1X protease inhibitor mixture (Roche). Lysates were clarified by centrifugation for 20 min at 14,000 rpm and supernatants were collected. Protein concentration in the soluble fraction was determined by BCA protein assay (ThermoFisher Scientific). Proteins were separated by electrophoresis through 10% Tris-glycine gels (Bio-Rad) and transferred to nitrocellulose membrane. Primary antibodies used for western blot analysis were mouse monoclonal anti-NF-YA (Santa Cruz Biotechnology, sc-10779) at 1:500 dilution and rabbit polyclonal anti-α Tubulin (Abcam, ab18251) at 1:1000 dilution. Secondary antibodies used were horseradish peroxidase-conjugated goat anti-rabbit IgG (1:5000 dilution). The blot was then developed using SuperSignal West Femto kit (ThermoFisher Scientific) and images were acquired with a ChemiDoc XRS+ Imaging (BioRad) according to the manufacturer's instructions.

Chromatin Immunoprecipitation

Cells were cultured to ~70-80% confluency in five 150 mm plates. Cells were fixed with 1% formaldehyde in serum-free media for 10 min at RT on a shaker. Formaldehyde crosslinking was quenched with a final concentration of 125 mM glycine. Cells on each plate were then washed in cold 1X PBS and scraped in 5 ml of 1X PBS. Cells were then pelleted by spinning at ~5,000 rpm for 5 min and lysed with 4 ml cell lysis buffer (5 mM PIPES, pH 8.0, 85 mM KCl, 0.5% NP-40). After incubating on ice for 5 min, the nuclei were washed with cell lysis buffer and spun down again. The nuclei were then lysed by nuclei lysis buffer (50 mM Tris-HCl, pH 8.1, 10 mM EDTA, 1% SDS) and incubated on ice for 10 min. The nuclear extract was transferred into the TPX plastic microtubes (Diagenode) for sonication. The nuclear lysate was then sonicated in a Bioruptor sonicator (Diagenode) at high settings for 20-minute sonication cycle, 30sec ON/30sec OFF at 4 ºC to yield DNA fragments with an average length of ~500 bp. The sonicated lysate was centrifuged at 14,000 rpm for 10 min at 4 ºC and the supernatant was collected as chromatin extract. The chromatin extract (15-25µg) was diluted 10-fold and pre-cleared with Protein A/G Dynabeads (ThermoFisher Scientific) for 15 min at 4 ºC. Antibodies including anti-H3K4me2 (Abcam, ab32356, 6 µl), anti-H3K4me3 (Abcam, ab8580, 2 µl), anti-H3K79me2 (Abcam, ab3594, 2 µl) and anti-H3 (Abcam, ab1791, 2 µl) were added separately to the precleared chromatin extract and incubated overnight. Rabbit IgG antibody (2 µl) was added to a separate sample as the negative control. Fresh Protein A/G Dynabeads were then added for 3 hours at 4 ºC to immunoprecipitate chromatin and beads were sequentially washed in low salt, high salt, LiCl and TE buffer. DNA/protein complexes were eluted and treated with RNase A and crosslinking was reversed by heating overnight at 65°C. Samples were subsequently treated with Proteinase K for 2 hours. DNA was extracted using Qiaquick PCR purification kit (Qiagen)

and eluted using 100 µl water. Each qPCR reaction was performed in duplicate in 20 µl reaction volumes containing 2 µl of ChIP-enriched or input DNA, iTaq™ Universal SYBR Green Supermix (Bio-Rad), and region-specific primer sets. Target amplification and detection were performed in a CFX-96 Real-Time PCR Detection System (Bio-Rad). Primer sequences are listed in Supplementary Table S4.

Supplementary References

1 Padovan-Merhar, O. *et al.* Single mammalian cells compensate for differences in cellular volume and DNA copy number through independent global transcriptional mechanisms. *Mol Cell* **58**, 339-352, doi:10.1016/j.molcel.2015.03.005 (2015).

2 Kempe, H., Schwabe, A., Cremazy, F., Verschure, P. J. & Bruggeman, F. J. The volumes and transcript counts of single cells reveal concentration homeostasis and capture biological noise. *Mol Biol Cell* **26**, 797-804, doi:10.1091/mbc.E14-08-1296 (2015).

3 Potter, C. J. & Luo, L. Splinkerette PCR for mapping transposable elements in Drosophila. *PLoS One* **5**, e10168, doi:10.1371/journal.pone.0010168 (2010).

4 Chen, C. Y., Ezzeddine, N. & Shyu, A. B. Messenger RNA half-life measurements in mammalian cells. *Methods Enzymol* **448**, 335-357, doi:10.1016/S0076-6879(08)02617-7 (2008).

5 Heintz, N., Sive, H. L. & Roeder, R. G. Regulation of human histone gene expression: kinetics of accumulation and changes in the rate of synthesis and in the half-lives of individual histone mRNAs during the HeLa cell cycle. *Molecular and cellular biology* **3**, 539-550 (1983).

6 Martin, R. M., Rino, J., Carvalho, C., Kirchhausen, T. & Carmo-Fonseca, M. Live-cell visualization of pre-mRNA splicing with single-molecule sensitivity. *Cell reports* **4**, 1144-1155, doi:10.1016/j.celrep.2013.08.013 (2013).

Suppl. Figure S1

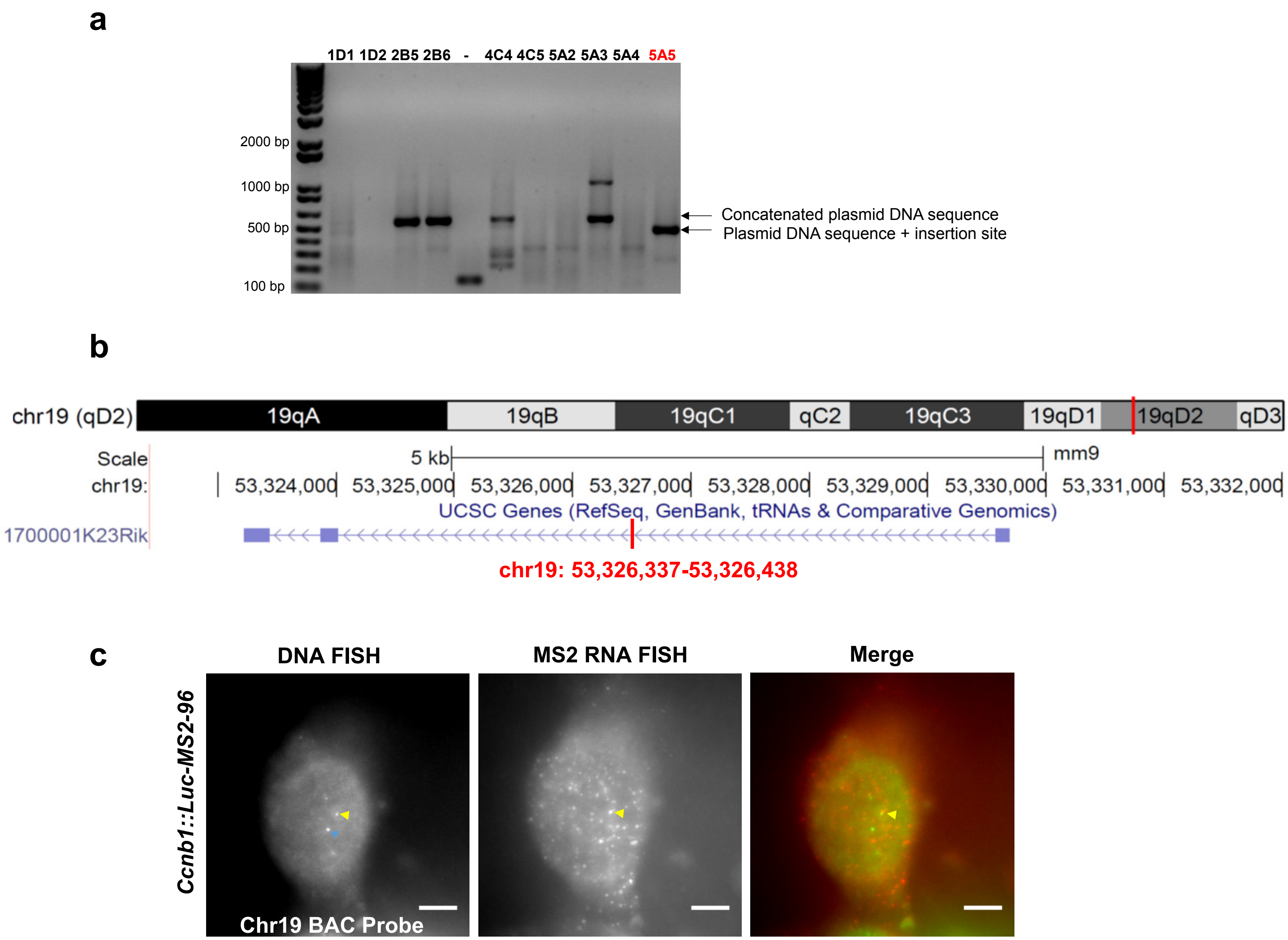

Supplementary Figure S1. Generating a stable Flp-In C2C12 cell line for site-specific integration of single-copy promoter transgenes. (a) An agarose gel image of splinkerette PCR products. The upper arrow indicates PCR products of identical sizes from several cell lines that contain concatenated *pFRT/LacZeo* plasmid DNA sequence. The lower arrow in the Flp-In cell line 5A5 indicates PCR product containing *pFRT/LacZeo* plasmid DNA sequence linked to the insertion site DNA sequence at the ScaI restriction site. PCR products were sequenced to identify the insertion sites. **(b)** UCSC Genome Browser view of the insertion site of the *pFRT/LacZeo* vector in C2C12 Flp-in cell line 5A5. The insertion site (marked in vertical red lines) was found to be located within the first intron of a non-coding RNA gene (*1700001K23Rik*) on mouse chromosome 19. **(c)** Representative RNA-DNA FISH images of cells containing the *Ccnb1::Luc-MS2* transgene (clone 96). RNA FISH probes target MS2 RNA and DNA FISH probes target the genomic DNA sequence at the integration site at chromosome 19. Merged images are shown on the right. Yellow arrowheads indicate the transgene loci and the cyan arrowhead indicates the other allele without the integrated transgene. Scale bars: 5 µm.

Suppl. Figure S2

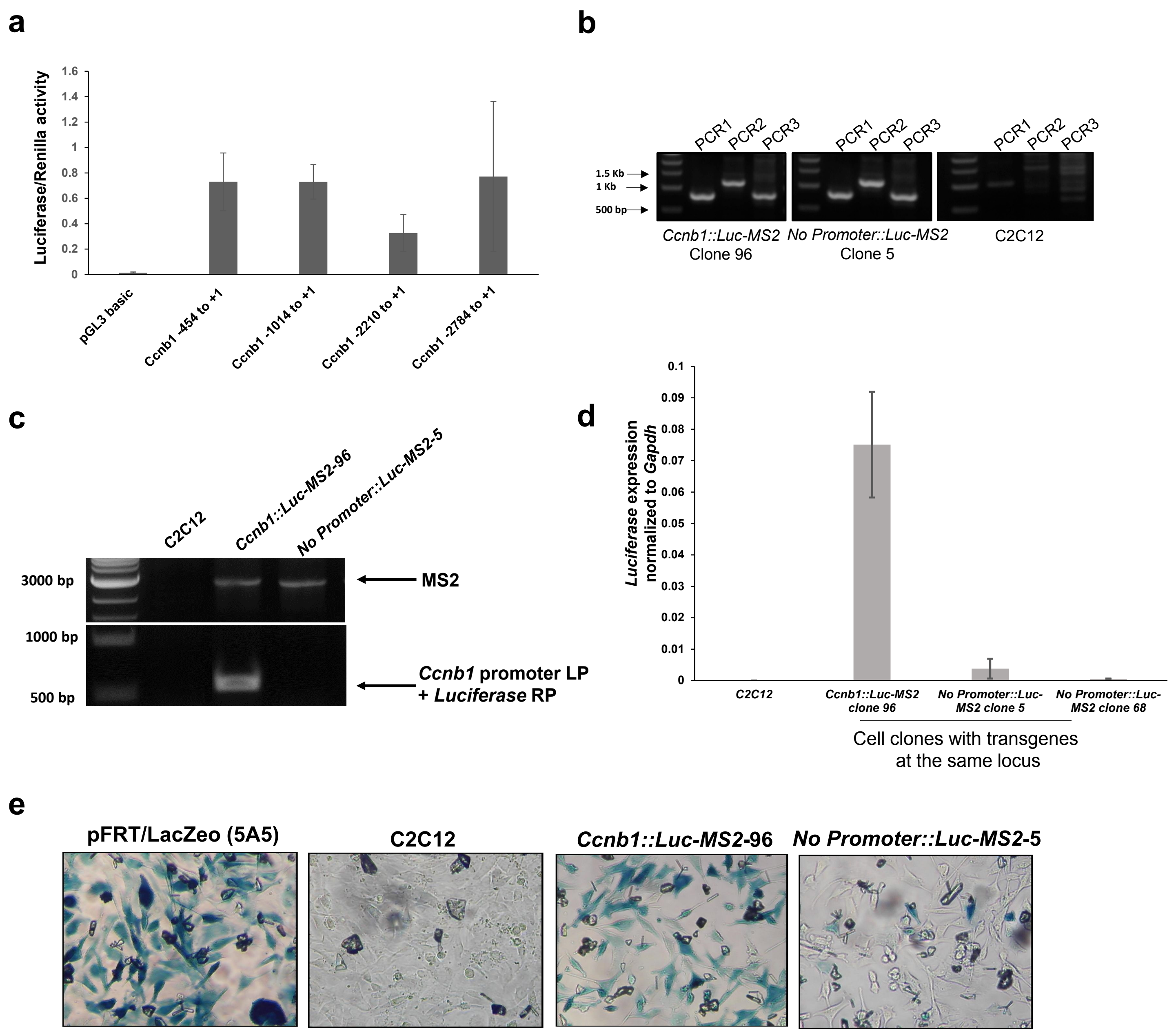

Supplementary Figure S2. Characterizing C2C12 cell clones with integrated single-copy transgenes driven by the *Ccnb1* promoter or without a promoter. (a) Dual-Glo luciferase reporter assay to measure activities of various mouse *Ccnb1* promoter fragments. Mouse *Ccnb1* promoter fragments (+1 to -454, -1014, -2210 and -2784) were cloned in the pGL3-Basic vector. pRenilla vector was co-transfected to normalize for transfection efficiency. Error bars represent standard deviations (n = 2). **(b)** Agarose gel images of genomic DNA PCR to verify transgene integration in these two cell clones: *Ccnb1::Luc-MS2* (clone 96) and *No Promoter::Luc-MS2* (clone 5). Primer locations are shown in Figure 1. Genomic DNA from wildtype C2C12 cells served as negative controls. **(c)** Agarose gel images of genomic DNA PCR to detect the presence of MS2 repeats and the *Ccnb1* promoter in *Ccnb1::Luc-MS2* (clone 96) cells and *No Promoter::Luc-MS2* (clone 5) cells. **(d)** *Luciferase* mRNA expression in cells containing *Ccnb1::Luc-MS2* (clone 96) and *No Promoter::Luc-MS2* (clone 5 and clone 68) detected by RT-qPCR. Wildtype C2C12 cells served as a negative control. **(e)** *β*-Gal staining images of the following cell clones: pFRT/lacZeo Flp-In cell line (clone 5A5), wild-type C2C12, *Ccnb1::Luc-MS2* (clone 96) and *No Promoter::Luc-MS2* (clone 5).

Suppl. Figure S3

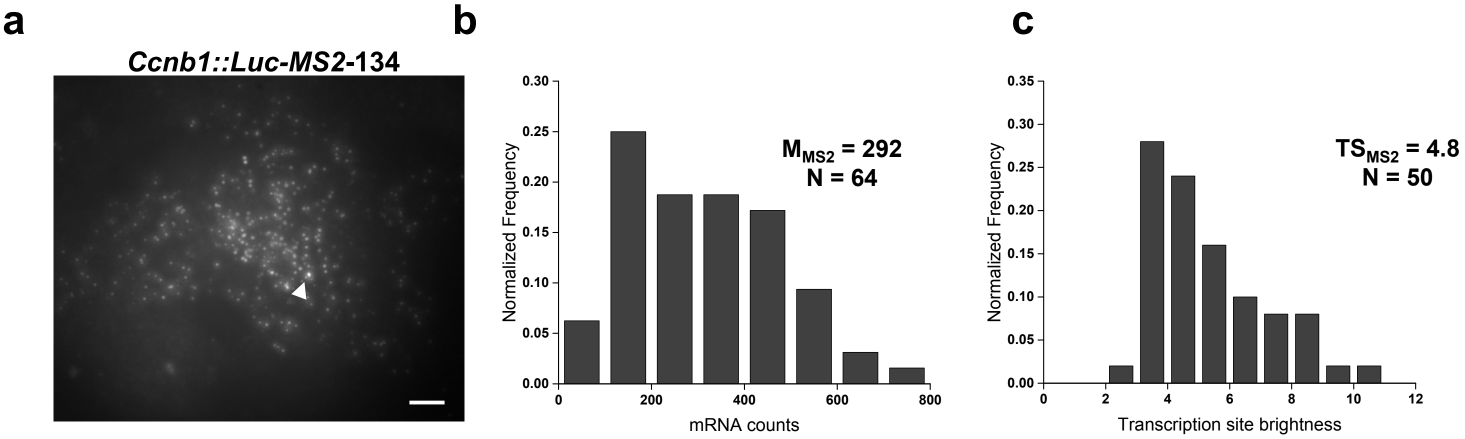

Supplementary Figure S3. Quantifying mRNA counts and transcription site brightness from an additional cell clone containing the *Ccnb1::Luc-MS2* transgene (clone 134). (a) Representative single molecule RNA FISH images of *Ccnb1::Luc-MS2* (clone 134) using MS2 FISH probes (120X magnification). The white arrow indicates the transcription site. Scale bar: 5 µm. **(b)** Histograms of mRNA counts of the *Ccnb1::Luc-MS2* transgene**.** M_{MS2} indicates the median mRNA count of the *Ccnb1::Luc-MS2* transgene measured by MS2 FISH. N indicates the measured cell number. **(c)** Histograms of transcription site (TS) brightness of the *Ccnb1::Luc-MS2* transgene. TS_{MS2} indicates the median TS brightness of the *Ccnb1::Luc-MS2* transgene measured by MS2 FISH. N indicates the measured TS number. FISH images from one experiment were analysed.

Suppl. Figure S4

a

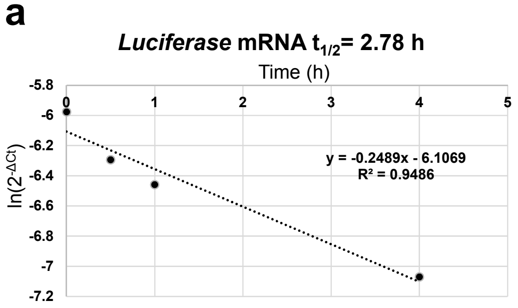

b

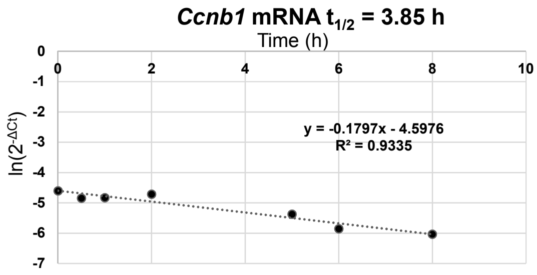

c

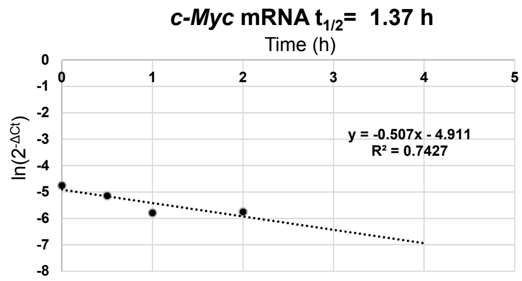

d

mRNA $t_{1/2}$	Mean
Luciferase	2.49 ± 0.29 h
Ccnb1	3.83 ± 0.04 h
c-Myc	1.01 ± 0.32 h

Supplementary Figure S4. Measuring decay kinetics of *Luciferase, Ccnb1* and *c-Myc* mRNA in the *Ccnb1::Luc-MS2* cell clone. (a–c) Representative mRNA decay curves of *Luciferase*, native *Ccnb1* and *c-Myc* in the *Ccnb1::Luc-MS2* (clone96) cells after inhibiting transcription with 30 µg/ml DRB. *18S* rRNA was used to normalize the expression. In the mRNA decay curves, $ln(2^{-(Ct_{mRNA}-Ct_{18S})})$ values were plotted in the y-axis and the corresponding time points were plotted in the x-axis. mRNA half-lives were calculated as described in Supplementary Methods. **(d)** Table showing calculated average half-lives ($t_{1/2}$) of *Luciferase*, *Ccnb1* and *c-Myc* mRNA (n= 3, 2 and 3, respectively). Errors represent standard deviations.

Suppl. Figure S5

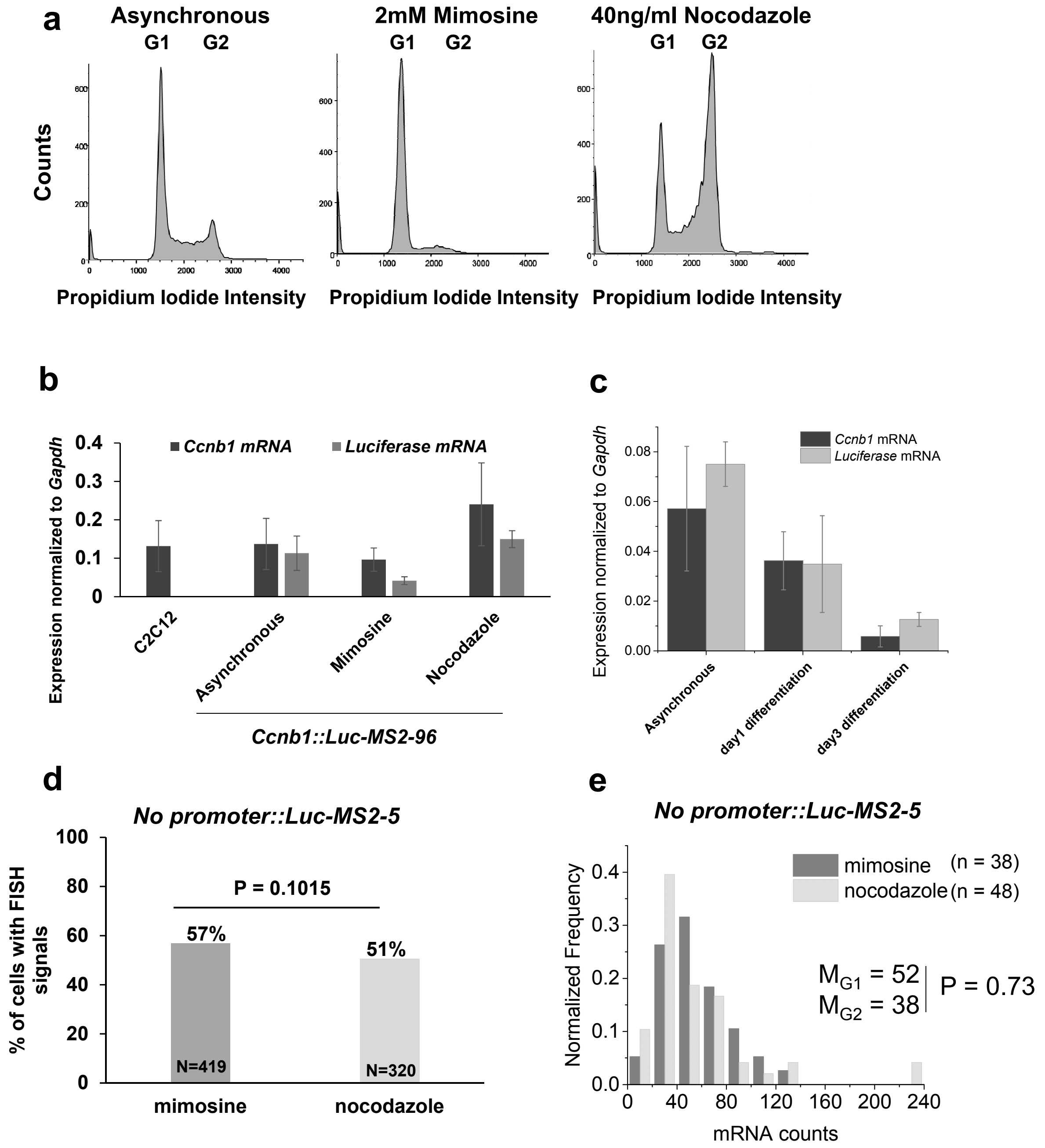

Supplementary Figure S5. Effects of cell cycle arrest on *Ccnb1::Luc-MS2* transgene expression and *No promoter::Luc-MS2* transgene expression. **(a)** Cell cycle profiles of asynchronous cells, mimosine-treated cells (G1 arrest) and nocodazole-treated cells (G2/M arrest) by flow cytometry. **(b)** Measuring native *Ccnb1* mRNA and *Luc-MS2* mRNA levels in asynchronous cells and cells arrested at G1 or G2/M by RT-qPCR. mRNA expression was normalized to *Gapdh* mRNA levels. Results are averages from three independent repeats. Error bars are standard deviations. **(c)** Measuring native *Ccnb1* gene and *Ccnb1::Luc-MS2* transgene expression upon induction of myogenic differentiation by RT-qPCR. mRNA expression was normalized to *Gapdh* mRNA levels. Error bars are standard deviations (n = 2). **(d)** Fractions of the *No Promoter::Luc-MS2* transgene cells displaying MS2 FISH signals after being arrested by mimosine (G1) or nocodazole (G2/M). P-values were determined by Fisher's exact test. **(e)** Histograms and medians of mRNA counts of cells containing the *No Promoter::Luc-MS2* transgene after being arrested by mimosine (G1) or nocodazole (G2/M). mRNA counts were not significantly different between G1-arrested cells and G2/M-arrested cells as determined by students' t-test. 40X (multiple cell) and 120X (single cell) FISH images from two experiments were analysed.

Suppl. Figure S6

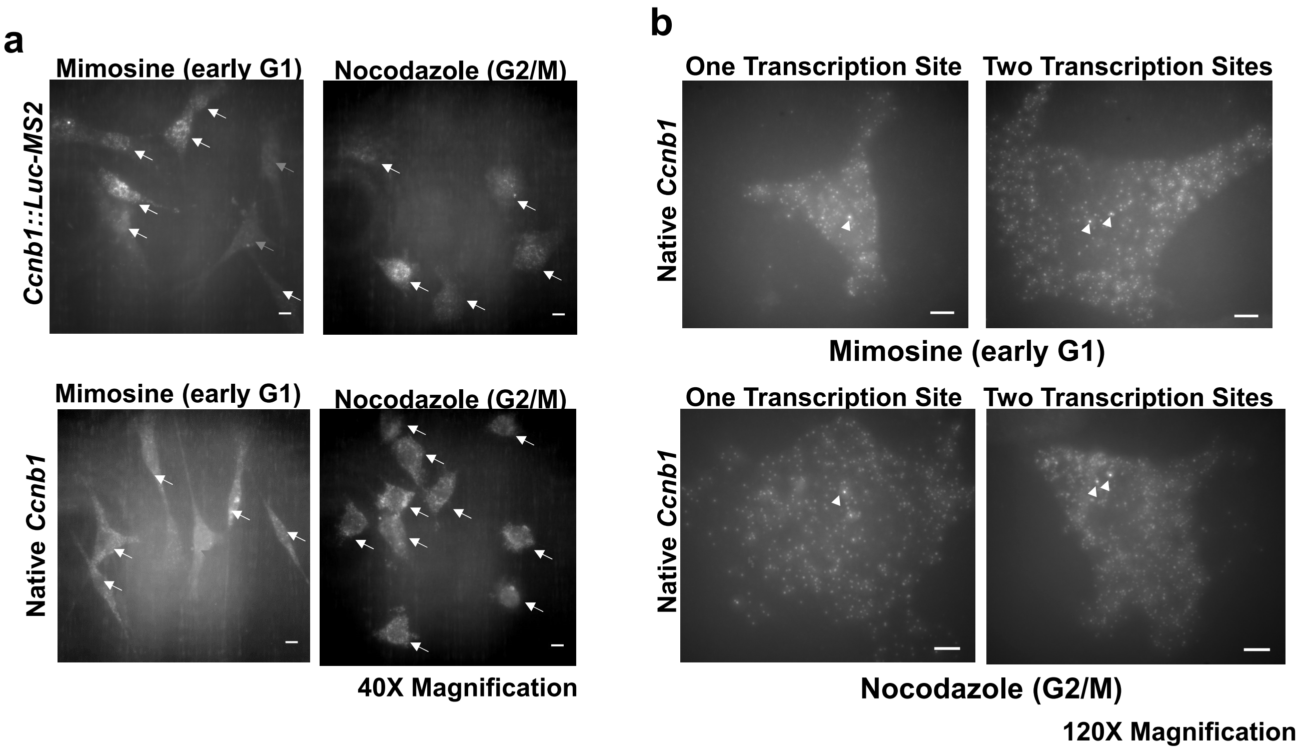

Supplementary Figure S6. Expression of the *Ccnb1::Luc-MS2* transgene and number of active native *Ccnb1* alleles in G1 and G2/M. (a) Representative single molecule RNA FISH images of the *Ccnb1::Luc-MS2* transgene and native *Ccnb1* mRNA in mimosine-treated and nocodazole-treated *Ccnb1::Luc-MS2* cells (clone 96), respectively. Images were taken at 40X magnification. White arrows indicate cells with FISH signals. Grey arrows indicate cells without FISH signals. Scale bars: 10 µm. **(b)** Representative single molecule RNA FISH images of the native *Ccnb1* gene in *Ccnb1::Luc-MS2* cells (clone 96) treated with mimosine and nocodazole, respectively. Images were taken at 120X magnification. White arrowheads indicate the TSs. Scale bars: 5 µm.

Suppl. Figure S7

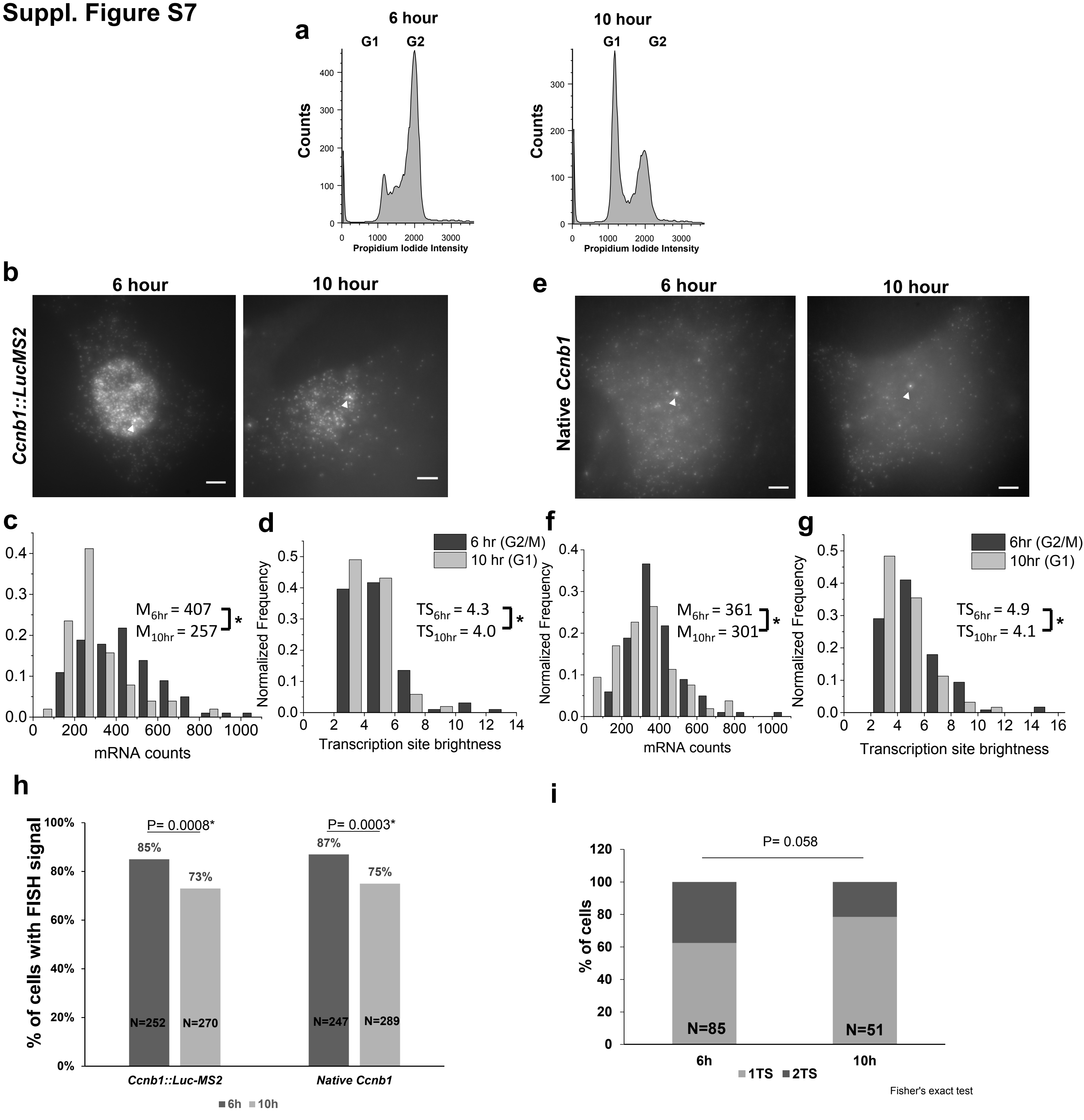

Supplementary Figure S7. Expression of the *Ccnb1::Luc-MS2* transgene and the native *Ccnb1* gene in cells synchronized at G1 or G2/M measured by single molecule RNA FISH. **(a)** Flow cytometry profiles of cells synchronized with thymidine/aphidicolin and released for 6 hours and 10 hours. **(b)** Representative single molecule RNA FISH images using MS2 probes in *Ccnb1::Luc-MS2* (clone-96) cells synchronized at G2/M (6 hours) or at G1 (10 hours). White arrowheads indicate the TSs. Scale bars: 5 µm. **(c, d)** Histograms and medians of mRNA counts and TS brightness of the *Ccnb1::Luc-MS2* transgene. Asterisks (*) indicate statistically significant differences between G2/M cells and G1 cells. $p < 1e-4$ in panel C (n= 101 and 51 for G1 and G2/M cells, respectively). $p = 0.03$ in panel D (n = 96 and 51 for G1 and G2/M cells, respectively). **(e)** Representative single molecule RNA FISH images of native *Ccnb1* mRNA in *Ccnb1::Luc-MS2* (clone 96) cells synchronized at G2/M (6h) or at G1 (10h). White arrowheads indicate the TSs. Scale bars: 5 µm. **(f, g)** Histograms and medians of mRNA counts and TS brightness of the native *Ccnb1* gene. Asterisks (*) indicate statistically significant differences between G2/M cells and G1 cells. $p = 0.005$ in panel F (n = 101 and 53 for G1 and G2/M cells, respectively). $p = 0.005$ in panel G (n = 117 and 62 for G1 and G2/M cells, respectively). In panels C, D, F and G, student's t test was used to determine statistically significant differences. **(h)** Fractions of cells with *Luc-MS2* FISH signals and native *Ccnb1* FISH signals in *Ccnb1::Luc-MS2* cells synchronized at G2/M (6h) or at G1 (10h). Fisher's exact test was used to determine the statistical significance. Asterisks (*) indicate statistically significant differences ($p < 0.05$). **(i)** Fractions of cells with one or two TSs of the native *Ccnb1* gene among cells synchronized at G2/M (6h) or at G1 (10h). Fisher's exact test was used to determine the P value. FISH images from one successful cell cycle synchronization experiment were analysed.

Suppl. Figure S8

10 hr post release (G1) | **6 hr post release (G2/M)**

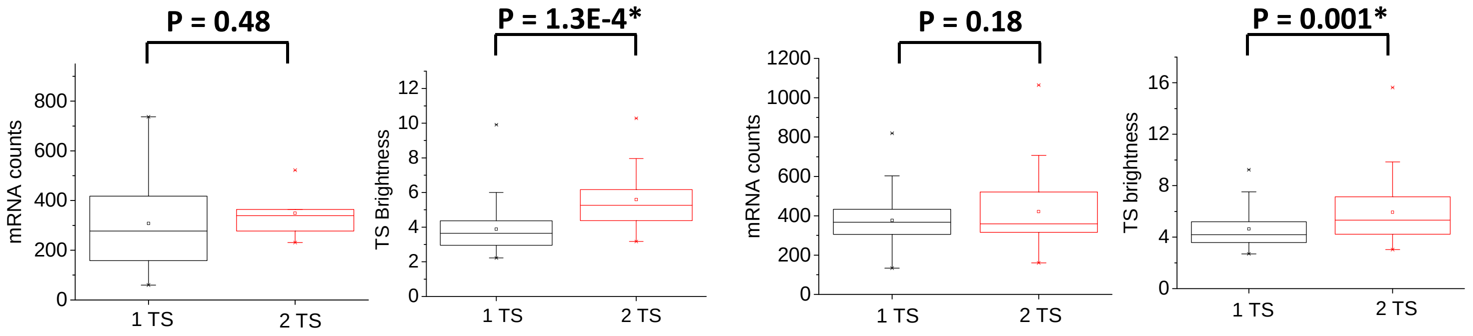

10 hr post release (G1)

M_{1TS} = 278 (n=40)
M_{2TS} = 339 (n=11)

TS_{1TS} = 3.6 (n=40)
TS_{2TS} = 5.3 (n=22)

	One TS	Two TS
**Pol II density**	1.21 kb^{-1}	1.91 kb^{-1}
**Pol II firing rate**	1.34 $\pm$ 0.19 min^{-1}	2.11 $\pm$ 0.30 min^{-1}

6 hr post release (G2/M)

M_{1TS} = 367 (n=53)
M_{2TS} = 359 (n=31)

TS_{1TS} = 4.2 (n=53)
TS_{2TS} = 5.3 (n=62)

	One TS	Two TS
**Pol II density**	1.46 kb^{-1}	1.91 kb^{-1}
**Pol II firing rate**	1.61 $\pm$ 0.23 min^{-1}	2.11 $\pm$ 0.30 min^{-1}

Supplementary Figure S8. mRNA counts, TS brightness and transcription rates from cells synchronized at G1 or G2/M with one or two active *Ccnb1* alleles. Box plots show mRNA counts or TS brightness of the native *Ccnb1* gene among cells synchronized at G1 or G2/M with 1 TS or 2 TS. Measured cell numbers and TS numbers are noted below each box plot. Asterisks (*) indicate statistically significant differences between the two experimental groups determined by student's t-test (P values are noted in the plots). Tables show calculated Pol II densities and Pol II firing rates of the native *Ccnb1* gene among cells synchronized at G1 or G2/M with 1 TS or 2 TS, respectively.

Suppl. Figure S9

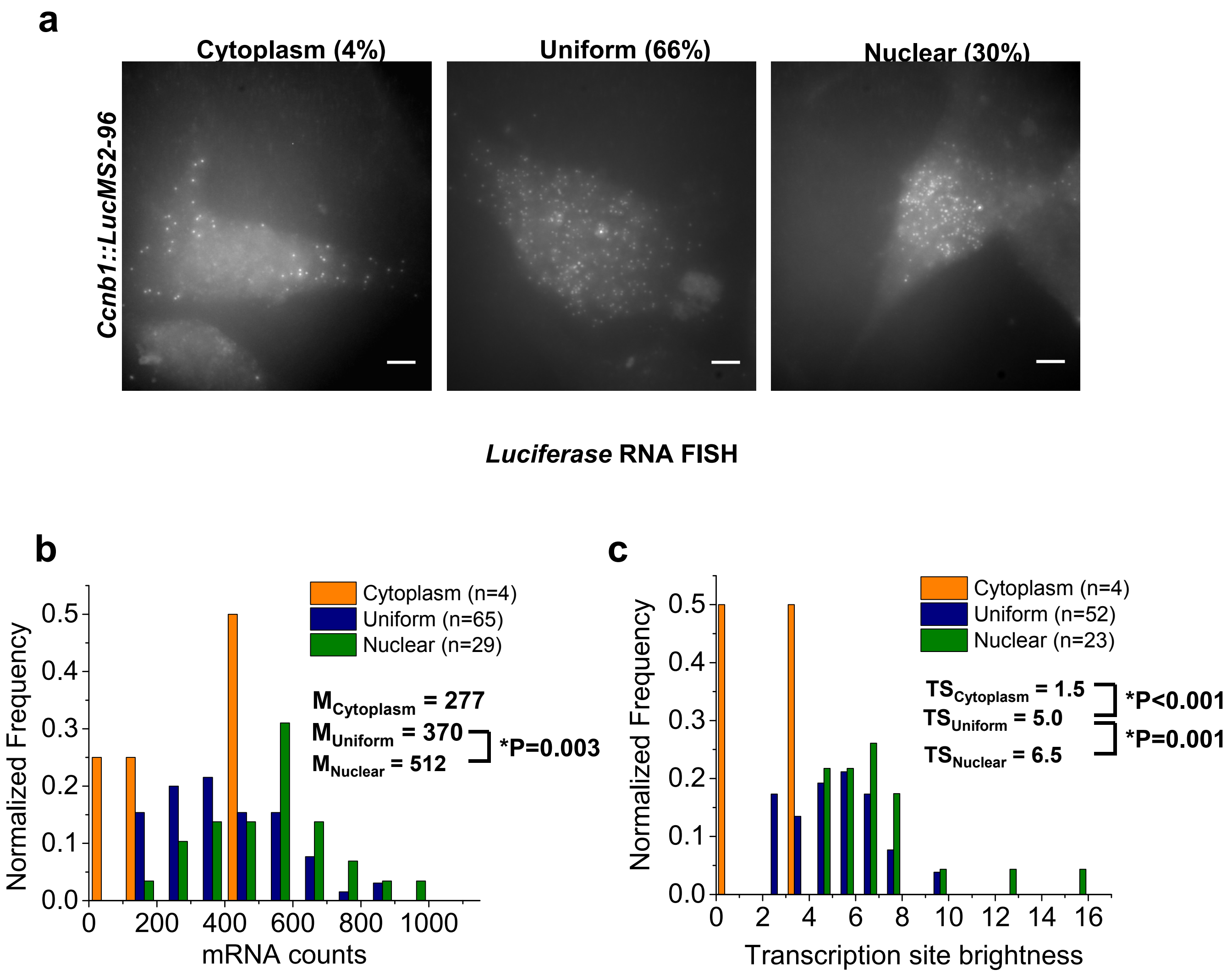

Supplementary Figure S9. Distinct subcellular localizations of *Luc-MS2* mRNA detected by *Luciferase* RNA FISH. (a) Representative images of single molecule RNA FISH using probes against *Luciferase* mRNA in asynchronous *Ccnb1::Luc-MS2* (clone 96) cells. Cells display cytoplasmic, uniform or nuclear localization of *Luc-MS2* mRNA. The percentages of cells with respective subcellular localizations of *Luc-MS2* mRNA are shown in parentheses (N = 98). Scale bars: 5 µm. **(b, c)** Histogram and medians of mRNA counts (b) and TS brightness (c) of the *Ccnb1::Luc-MS2* transgene among cells exhibiting distinct subcellular mRNA localizations. Measured cell numbers and TS numbers are noted in the panels. Asterisks (*) indicate statistically significant differences between the two experimental groups determined by student's t-test (P values are noted in the panels). FISH images from one experiment were analysed.

Suppl. Figure S10

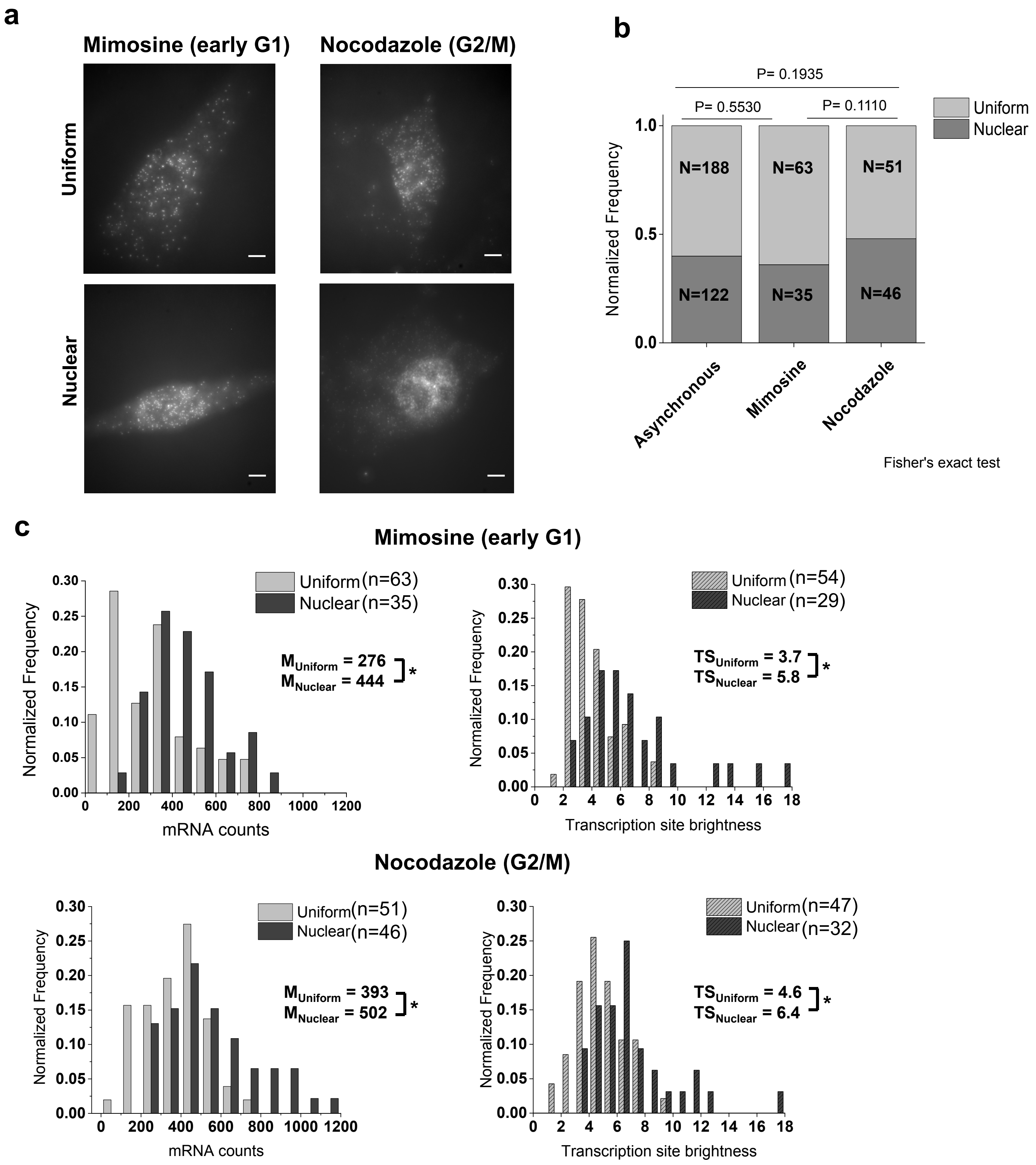

Supplementary Figure S10. Two cell populations differing in *Ccnb1* promoter activities and *Luc-MS2* mRNA localizations were found in cells arrested at G1 or G2/M. (a) Representative single molecule RNA FISH images of the *Ccnb1::Luc-MS2* transgene among cells treated with mimosine or nocodazole displaying nuclear- or uniformly-localized *Luc-MS2* mRNA. Images were taken at 120X magnification. Scale bars: 5 µm. **(b)** Fractions of cells with nuclear- or uniformly-localized *Luc-MS2* mRNA in asynchronous, mimosine-treated and nocodazole-treated cells, respectively**.** P values were determined by Fisher's exact test. Cells with cytoplasmic localized *Luc-MS2* mRNA were counted together with cells with uniformly localized *Luc-MS2* mRNA. **(c)** Histograms and medians of mRNA counts and TS brightness of the *Ccnb1::Luc-MS2* transgene among cells displaying uniformly- or nuclear-localized *Luc-MS2* mRNA in mimosine-treated or nocodazole-treated cells. Measured cell numbers N are shown in each plot. Asterisks (*) indicate statistically significant differences in mean values of mRNA counts or TS brightness among cells with nuclear- or uniformly-localized *Luc-MS2* mRNA determined by student's t-test ($p < 0.001$ in all cases). FISH images from one successful cell cycle arrest experiment were analyzed.

Suppl. Figure S11

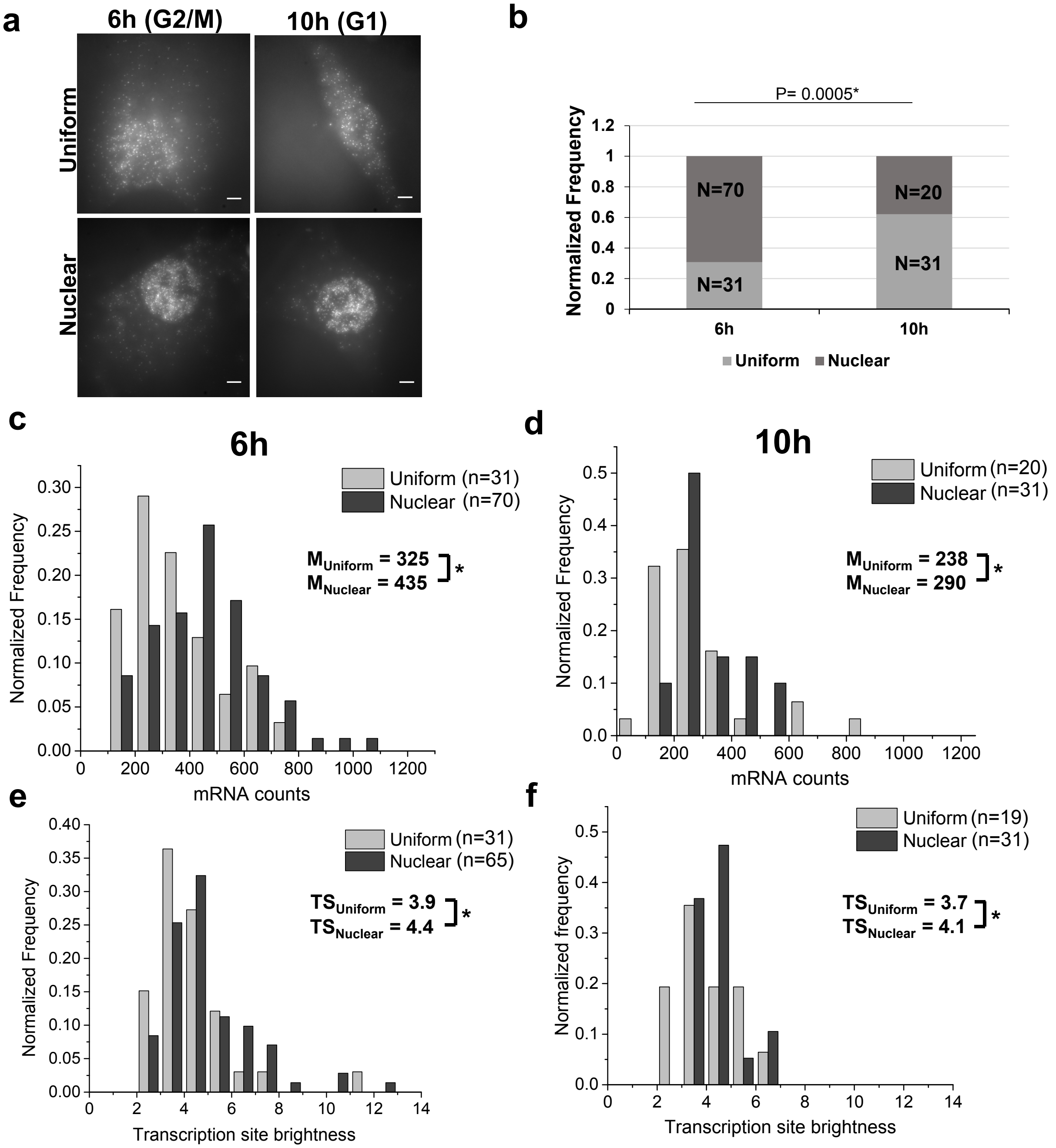

Supplementary Figure S11. Cells synchronized at G1 or G2/M also displayed distinct *Luc-MS2* mRNA localizations and *Ccnb1* promoter activities. (a) Representative single molecule RNA FISH images (MS2 FISH probes) in *Ccnb1::Luc-MS2* cells (clone 96) synchronized at G2/M (6h) or at G1 (10h) displaying nuclear- or uniformly-localized *Luc-MS2* mRNA. Images were taken at 120X magnification. Scale bars: 5 µm. **(b)** Fractions of cells displaying uniformly- or nuclear-localized *Luc-MS2* mRNA in synchronized cells. Asterisks (*) indicate statistically significant differences determined by Fisher's exact test ($p < 0.05$). **(c–f)** Histograms and medians of mRNA counts and TS brightness of the *Ccnb1::Luc-MS2* transgene among cells synchronized at G2/M (6h) or at G1 (10h) displaying uniformly- or nuclear-localized *Luc-MS2* mRNA. Measured cell and TS numbers are shown in the figure. Mann-Whitney U-test was used to compare mRNA counts or TS brightness among cells with uniformly- or nuclear-localized *Luc-MS2* mRNA. Asterisks (*) indicate statistically significant differences ($p < 0.05$ in all cases). FISH images from one successful cell cycle synchronization experiment were analysed.

Suppl. Figure S12

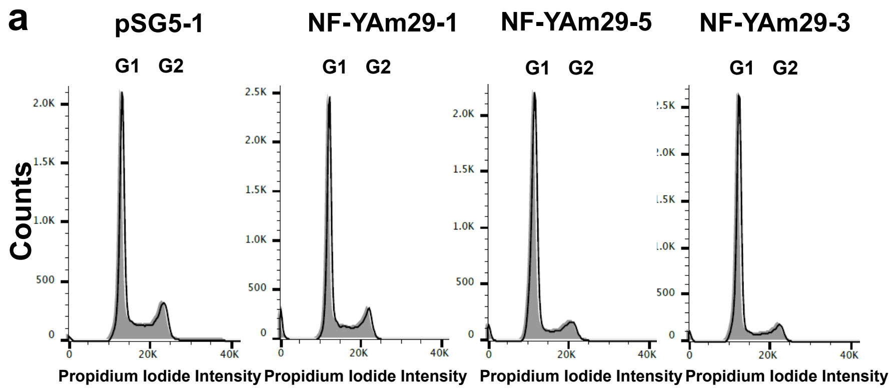

	pSG5-1	NF-YAm29-1	NF-YAm29-5	NF-YAm29-3
G1	66.7%	70%	78.6%	72.2%
S	13.3%	11.6%	9.0%	13.6%
G2	20%	18.3%	12.4%	12.7%

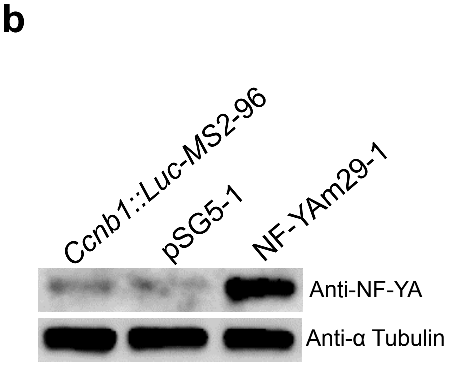

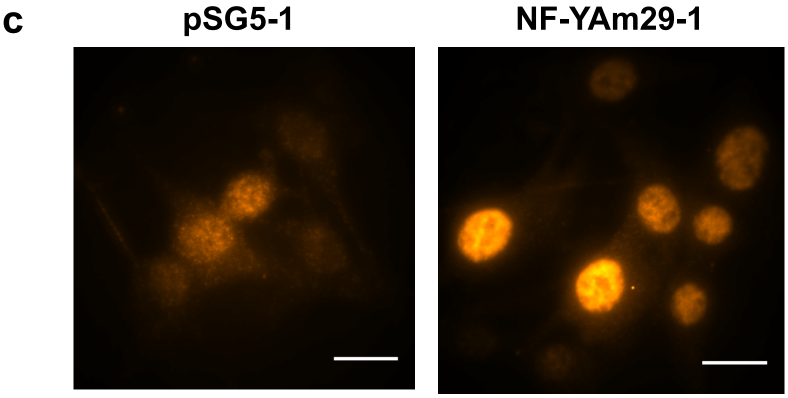

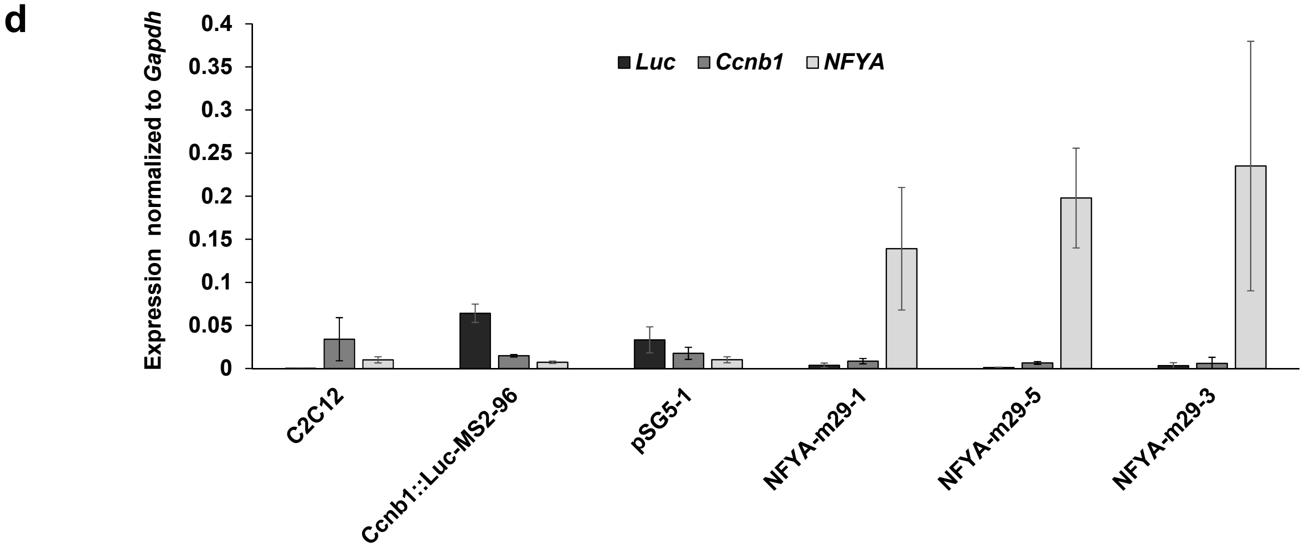

Supplementary Figure S12. Cell cycle profiles and mRNA expression in stable cell lines overexpressing a dominant negative mutant NF-YAm29. (a) Flow cytometry cell cycle profiles of *Ccnb1::Luc-MS2* (clone 96) cells stably transfected with the pSG5 vector (clone 1) or the pSG5NF-YAm29 vector (clone 1, clone 3 and clone 5). The percentages of cells in each cell cycle phase are presented in the table to the right. **(b)** Overexpression of NF-YA in the cell clone containing pSG5-NF-YAm29 vector (clone 1) was verified by western blot. Anti-α Tubulin was used as the loading control. **(c)** Detecting NF-YA expression in pSG5-1 control cells and NF-YAm29 (clone 1) cells by immunofluorescence staining. Greyscale images are shown in pseudocolor to provide contrast for fluorescence intensity. Scale bars: 20 μm. **(d)** Comparing levels of *Luciferase* mRNA, native *Ccnb1* mRNA and *NF-YA* mRNA in C2C12 cells, *Ccnb1::Luc-MS2* (clone 96) cells, pSG5 control cells (pSG5-1) and cell clones stably transfected with the pSG5-NF-YAm29 vector (clone 1, clone 3 and clone 5). mRNA expression was normalized to *Gapdh* mRNA levels. Error bars represent standard deviations (n=3).

Suppl. Figure S13

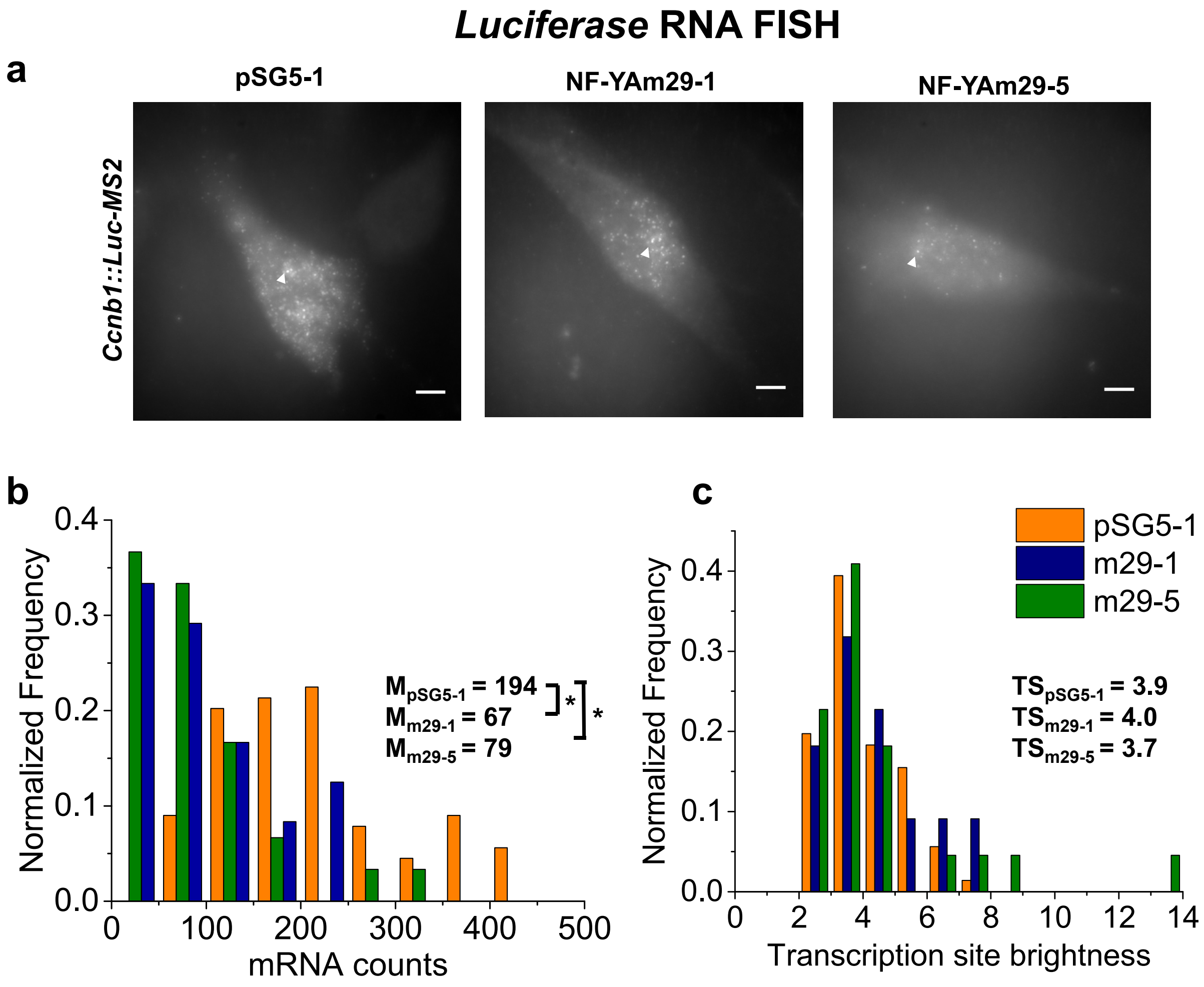

Supplementary Figure S13. Effects of expressing the NF-YAm29 mutant on *Ccnb1::Luc-MS2* transgene expression measured by RNA FISH using the *Luciferase* probe. (a) Representative single molecule RNA FISH images using *Luciferase* probes in cell clones with stably integrated pSG5-1 or pSG5-NF-YAm29 vectors (Clone 1 and Clone 5). Scale bars: 5 µm. Images were taken at 120X magnification. White arrowheads indicate the TSs. **(b)** Histograms and medians of mRNA counts. Measured cell numbers n = 89, 30 and 24 for pSG5-1, m29-1 and m29-5 cell clones, respectively. Asterisks (*) indicate statistically significant differences in mean values of mRNA counts between pSG5-1 cells and NF-YAm29-1 or NF-YAm29-5 cells determined by Student's t-test ($p < 1e\text{-}6$ in both cases). **(c)** Histograms and medians of TS brightness. Measured TS numbers n = 71, 22 and 22 for pSG5-1, m29-1 and m29-5 cell clones, respectively. TS brightness are not significantly different between pSG5-1 and NF-YAm29-1 or NF-YAm29-5 cells ($p = 0.34$ and 0.20). FISH images from one experiment were analysed.

Suppl. Figure S14

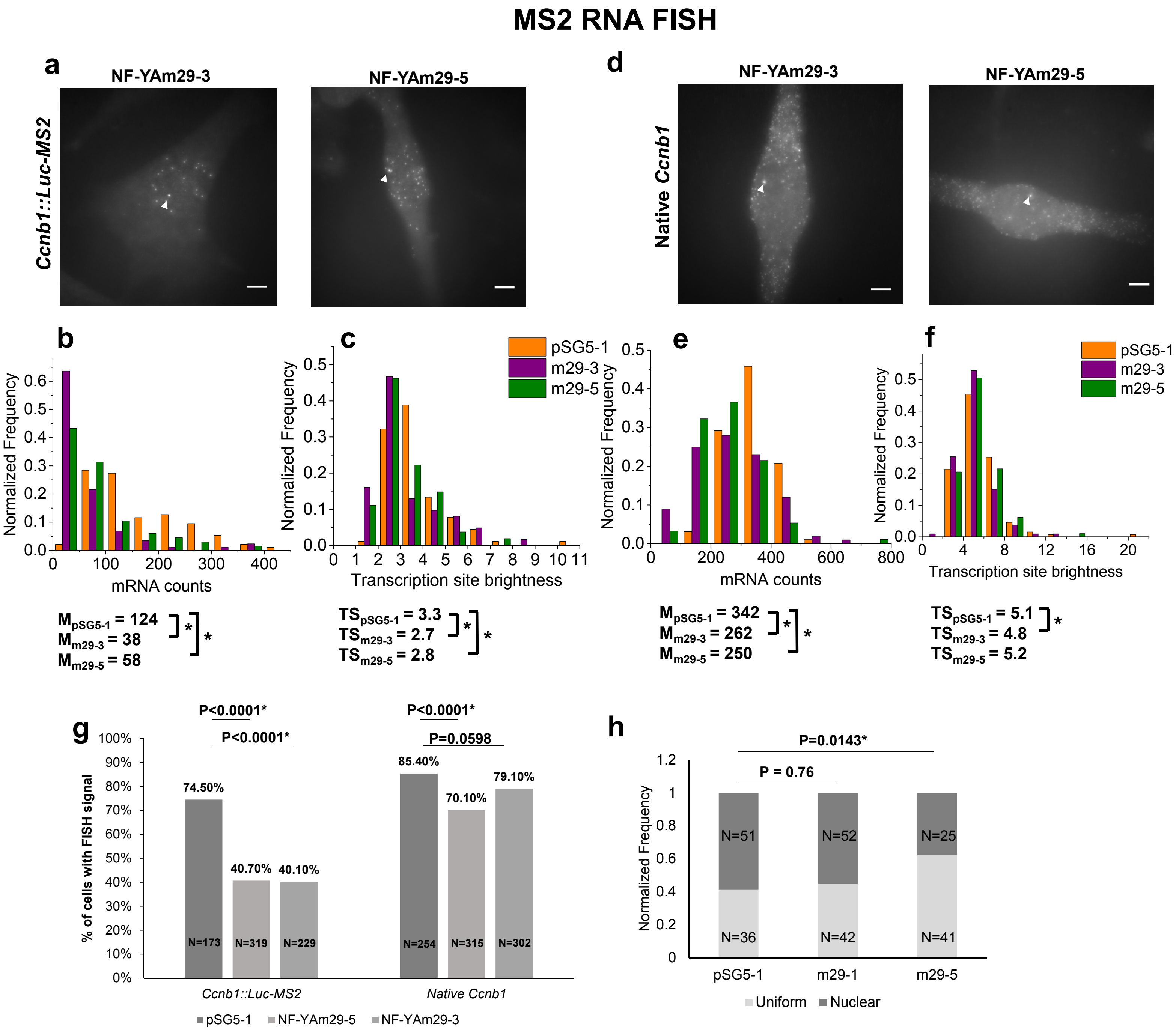

Supplementary Figure S14. Effects of expressing the NF-YAm29 mutant on *Ccnb1::Luc-MS2* transgene expression and native *Ccnb1* gene expression in additional cell clones. (a) Representative single molecule RNA FISH images using MS2 probes in cell clones stably transfected with the NF-YAm29 vector (NF-YAm29-3 and NF-YAm29-5). **(b)** Histograms and medians of mRNA counts of the *Ccnb1::Luc-MS2* transgene. Measured cell numbers N = 95, 88 and 67 for pSG5-1, m29-3 and m29-5 cell clone, respectively. **(c)** Histograms and medians of TS brightness of the *Ccnb1::Luc-MS2* transgene. Measured TS numbers N = 90, 62 and 54 for pSG5-1, m29-3 and m29-5 clone, respectively. **(d)** Representative single molecule RNA FISH images of native mouse *Ccnb1* mRNA in NF-YAm29-3 and NF-YAm29-5 cell clones. In panels A and D, images were taken at 120X magnification. White arrowheads indicate the TSs. Scale bars: 5 µm. **(e)** Histograms and medians of mRNA counts of the native *Ccnb1* gene. Measured cell numbers N = 96, 100 and 93 for pSG5-1, m29-3 and m29-5 clone, respectively. **(f)** Histograms and medians of TS brightness of the native *Ccnb1* gene. Measured TS numbers N = 130, 106 and 97 for pSG5-1, m29-3 and m29-5 clone, respectively. In panels b, c, e and f, student's t-test was used to determine statistically significant differences in mean values of mRNA counts or TS brightness between pSG5-1 cells and m29-3 (or m29-5) cells. Asterisk (*) indicates that the difference is statistically significant (in b, $p < 1e-6$; in c, $p = 0.02$ and 0.03; in e, $p < 1e-6$; in f, $p = 0.03$ between pSG5-1 and m29-3, $p = 0.64$ between pSG5-1 and m29-5). **(g)** Fractions of cells detected with *Luc-MS2* mRNA FISH signals and native *Ccnb1* mRNA FISH signals in the control cell clone pSG5-1 and cell clones stably expressing the NF-YAm29 mutant (m29-5 or m29-3). **(h)** Fractions of cells displaying uniformly- or nuclear-localized *Luc-MS2* mRNA in the control cell clone pSG5-1 and cell clones stably expressing the NF-YAm29 mutant (m29-1 or m29-5). In panels g and h, Fisher's exact test was used to determine the P

value. Asterisks (*) indicate statistically significant differences ($p < 0.05$). FISH images from one experiment were analysed.

Suppl. Figure S15

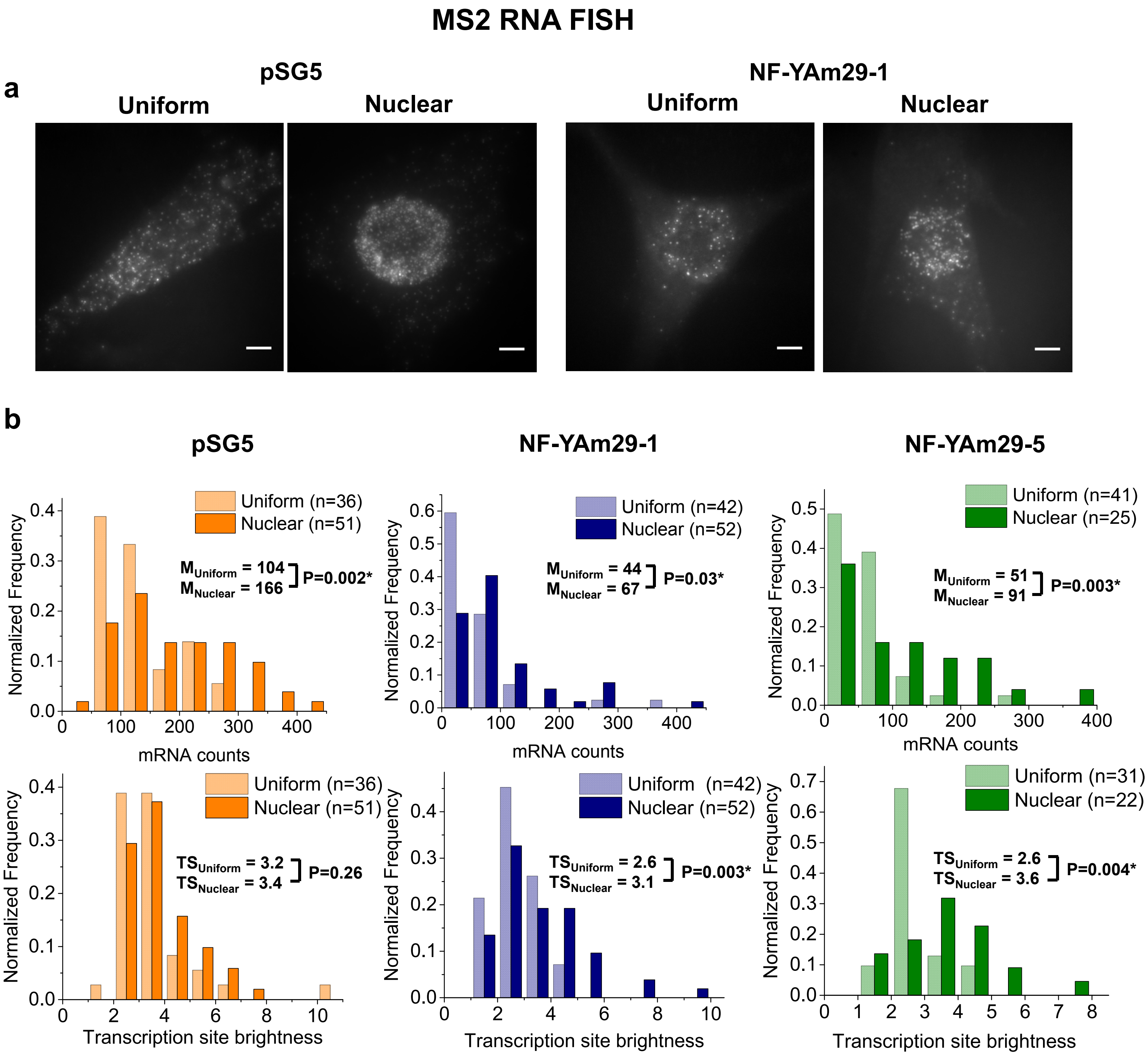

Supplementary Figure S15. Distinct *Luc-MS2* mRNA localizations and *Ccnb1* promoter activities in cells expressing the NF-YAm29 mutant. (a) Representative single molecule RNA FISH images of *Ccnb1::Luc-MS2* cells stably transfected with the control pSG5 vector (left) and the pSG5-NF-YAm29 vector (right) showing uniformly- or nuclear-localized *Luc-MS2* transcripts. Scale bars: 5 μm. **(b)** Histograms of mRNA counts and TS brightness in each stable cell line. Measured cell numbers n of each experimental group are shown in each plot. Asterisks (*) indicate statistically significant differences in mRNA counts or TS brightness between cells with uniformly- or nuclear-localized *Luc-MS2* mRNA determined by students' t-test ($p < 0.05$). FISH images from one experiment were analysed.

Suppl. Figure S16

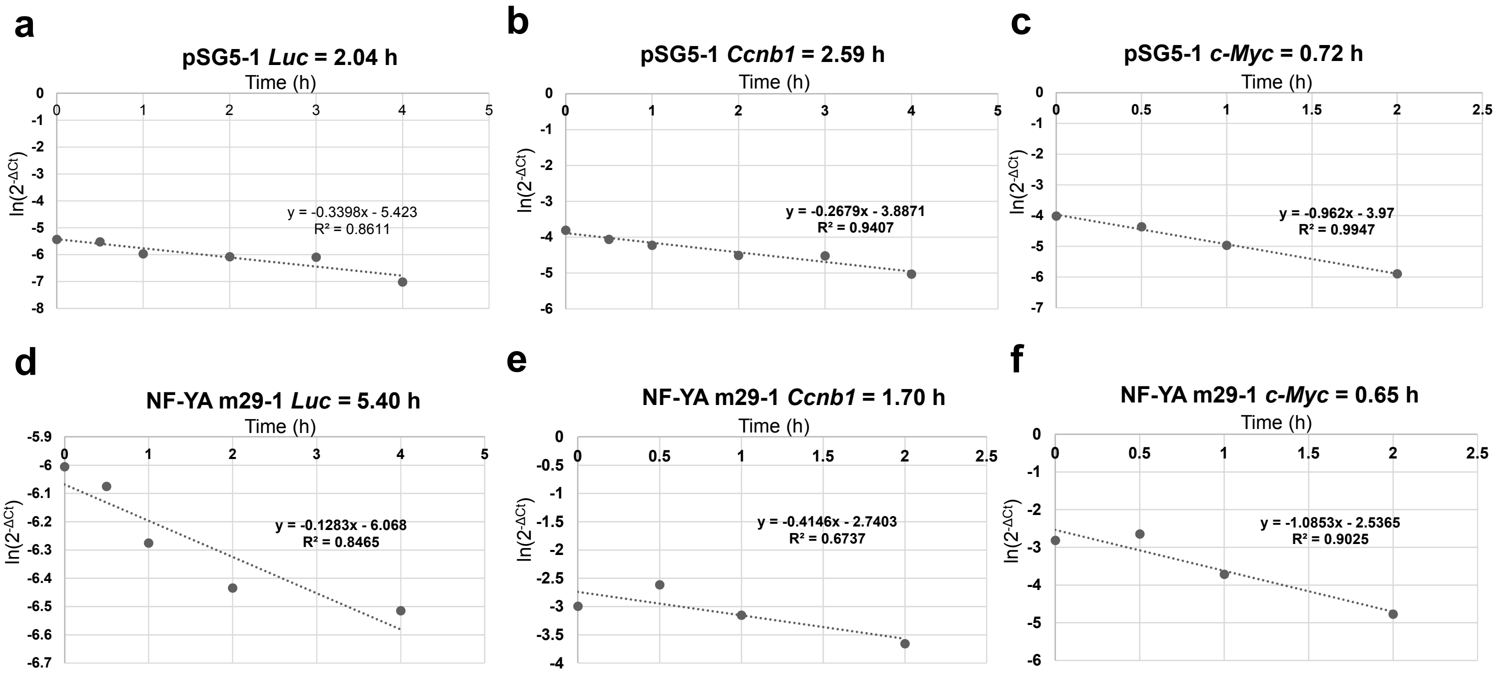

g

	mRNA $t_{1/2}$		mRNA decay rate k_{decay}	
	**pSG5-1**	**NF-YAm29-1**	**pSG5-1**	**NF-YAm29-1**
Luc	1.51 ± 0.70 h	5.8 ± 0.56 h	0.46 ± 0.15 h^{-1}	0.12 ± 0.012 h^{-1}
Ccnb1	2.40 ± 0.24 h	2.0 ± 0.42 h	0.29 ± 0.03 h^{-1}	0.35 ± 0.09 h^{-1}
c-Myc	0.65 ± 0.09 h	0.84 ± 0.27 h	1.07 ± 0.15 h^{-1}	0.82 ± 0.27 h^{-1}

Supplementary Figure S16. Comparing stability of *Luciferase, Ccnb1* and *c-Myc* mRNA in control cells and cell clones overexpressing NF-YAm29 mutant. (a–f) mRNA decay plots of *Luciferase*, native *Ccnb1* and *c-Myc* in cells stably transfected with the pSG5 vector (a–c) or the pSG5-NF-YAm29 vector (d–f), respectively. *18S* rRNA was used to normalize the expression. Decay curves were plotted and mRNA half-lives were calculated as described in Supplementary Methods. mRNA half-lives calculated from each mRNA decay curve are shown above each plot. **(g)** Table comparing average half-lives ($t_{1/2}$) and decay rates (k_{decay}) of *Luciferase* mRNA, native *Ccnb1* mRNA and *c-myc* mRNA from pSG5-1 and NF-YAm29-1 clones calculated from two biological replicates.

Suppl. Figure S17

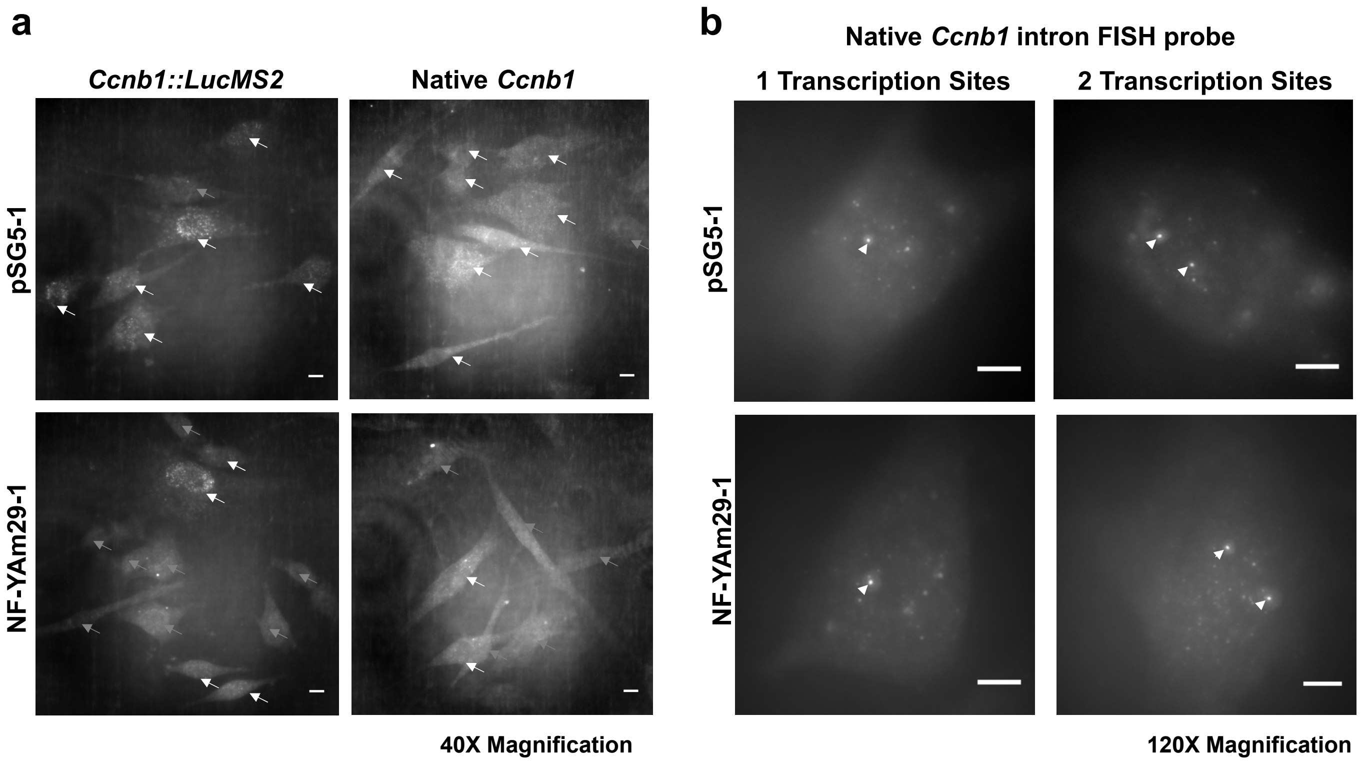

Supplementary Figure S17. Detecting the fraction of cells expressing the *Ccnb1::Luc-MS2* transgene and active TSs of the native *Ccnb1* gene in the presence of NF-YAm29 mutant. **(a)** Representative single molecule RNA FISH images of *Luc-MS2* mRNA (using MS2 probes) and native *Ccnb1* mRNA (using exon probes) in pSG5-1 and NF-YAm29-1 cell clones. Images were taken at 40X magnification. White and grey arrows indicate cells with and without MS2 FISH signals, respectively. Scale bars: 10 µm. **(b)** Representative single molecule RNA FISH images using probes targeting mouse *Ccnb1* introns 1 to 4 (see Supplementary Table S3) in pSG5-1 and NF-YAm29-1 cell clones. Images were taken at 120X magnification. White arrowheads indicate the TSs. Scale bars: 5 µm.

Suppl. Figure S18

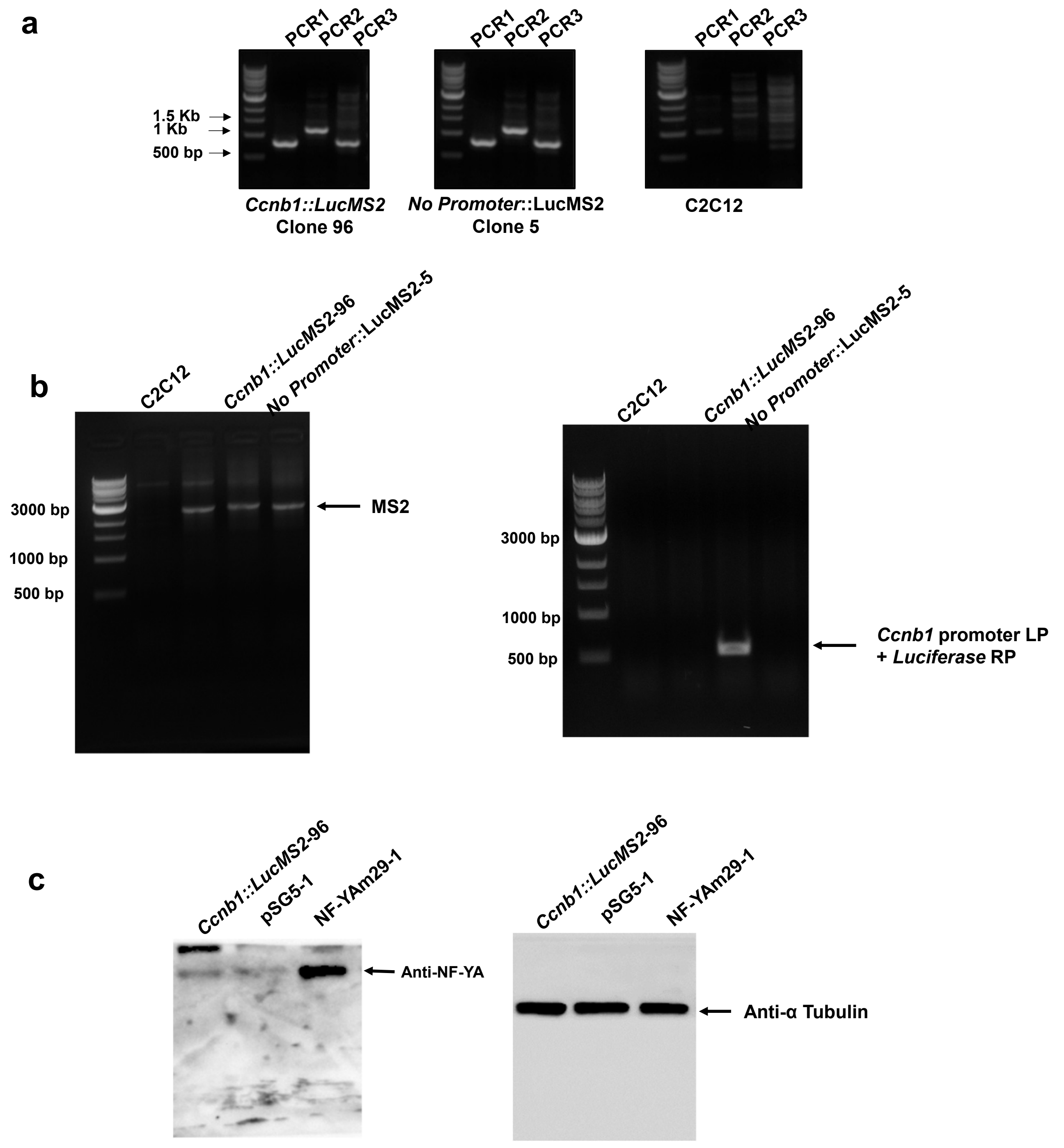

Supplementary Figure S18. Full-size gel images of Supplementary Figure S2b (a), S2c (b) and full-size western blot image of Supplementary Figure S12b (c).

Supplementary Tables

Supplementary Table S1. Sequences of single molecule FISH probes of mouse *Ccnb1* mRNA.

	**Probe**	**Position (bp)**	**Percent GC**
1	gctaaacactaacagccgtt	3	45.0%
2	cgattcgagaagacacccta	40	50.0%
3	tcctccaaaatcagaggttc	62	45.0%
4	attttcgtgttcctagtgac	97	40.0%
5	tggaagcagcagtaactgtc	171	50.0%
6	aatgtctccaagagcagttc	209	45.0%
7	aacagtaccttttccagtac	293	40.0%
8	cttctctacaggttttggta	320	40.0%
9	tggagagggattatcaacca	437	45.0%
10	gacacagatactcttctgca	486	45.0%
11	gtcactcactgcaaggatta	521	45.0%
12	attcactacagaggtttggg	564	45.0%
13	gaggatagctctcatgtttc	671	45.0%
14	cctgaagcagcctaaatttc	717	45.0%
15	ggacacagtcatgtacatgg	740	50.0%
16	gttctgcatgaaccgatcaa	764	45.0%
17	ataaacatggccgttacacc	817	45.0%
18	tacatctcctcatatttgct	841	35.0%
19	cacaaaggcgaagtcaccta	872	50.0%
20	gcttagtgtacgtgttgtta	894	40.0%
21	aatcttcatctccatctgtc	923	40.0%
22	ggctgaagttcagaactctg	945	50.0%
23	cgactttagatgctctacgg	993	50.0%
24	aggtatttggccaaagtgtg	1033	45.0%
25	gcaaaatgcaccatgtcgta	1075	45.0%
26	ttcagcgctaagcagaaagc	1117	50.0%
27	ccattcaccgttgtcaagaa	1139	45.0%
28	ggagtcttcactgtaggata	1181	45.0%
29	gttcaccatgactacattct	1229	40.0%
30	acagtcatgtgctttgtgag	1255	45.0%
31	cttagcatgcttagatgctg	1289	45.0%
32	tagtgtacagttcagctgtg	1322	45.0%
33	cacggccttagacaaattct	1346	45.0%
34	tctattggagttatgccttt	1368	35.0%
35	caactgcatctgcagatgta	1392	45.0%
36	gtaggtatcctatgtacagg	1425	45.0%
37	ccgttagcctaaactcagaa	1518	45.0%
38	cattagttggatccactgtt	1582	40.0%
39	ggaacaaaggactagctggt	1697	50.0%
40	gttaaaggtggcaaccactg	1723	50.0%
41	acccttatctgttgatagtg	1879	40.0%
42	tccttgggagtatatgcatg	1929	45.0%
43	acacacagtttgttcacact	2024	40.0%
44	agaatgcacttgaatcccac	2062	45.0%
45	taagaacactgtggagggca	2087	50.0%
46	catctggctgtcagaattca	2165	45.0%
47	attacaagacaggagtggcg	2209	50.0%
48	tgagacaggattcttttcca	2235	40.0%

Supplementary Table S2. Sequences of single molecule FISH probes of the 5'-portion of *Luciferase* mRNA.

	Probe	Position (bp)	Percent GC
1	catcttccagcggatagaat	39	45.0%
2	gtatctcttcatagccttat	80	35%
3	aagcaattgttccaggaacc	105	45.0%
4	taagtgatgtccacctcgat	139	45.0%
5	ccgaacggacatttcgaagt	167	50.0%
6	tatcgtttcatagcttctgc	190	40.0%
7	tctgtgatttgtattcagcc	212	40.0%
8	agttttcactgcatacgacg	234	45.0%
9	cccaacaccggcataaagaa	262	50.0%
10	cgcaactgcaactccgataa	290	50.0%
11	cgaaatgcccatactgttga	344	45.0%
12	aaacgaacaccacggtaggc	366	55.0%
13	aatttttgcaaccccttttt	389	30.0%
14	attgggagctttttttgcac	415	40.0%
15	cctggtaatccgttttagaa	459	40.0%
16	gacgaacgtgtacatcgact	485	50.0%
17	aaccgggaggtagatgagat	507	50.0%
18	ctctggcacaaaatcgtatt	533	40.0%
19	ttgtcttgtccctatcgaag	555	45.0%
20	gaggagttcatgatcagtgc	577	50.0%
21	tttaggcagaccagtagatc	599	45.0%
22	ttctatgaggcagagcgaca	621	50.0%
23	catgcgagaatctcacgcag	645	55.0%
24	aatcgcagtatccggaatga	692	45.0%
25	cattccaaaaccgtgatgga	728	45.0%

Supplementary Table S3. Sequences of single molecule FISH probes targeting intronic regions of the mouse *Ccnb1* gene.

	Probe	Percent GC
1	cttcaccattaagagcacag	45.0%
2	aaggcgaagaattcaccctg	50.0%
3	ataaaagcctccgtcgtaga	45.0%
4	ccaccctaaacatggtgaat	45.0%
5	aagcaagtttccacccaaat	40.0%
6	agcaagggtggtaggatcta	50.0%
7	tcacgggttaattaaggcgg	50.0%
8	taattgggctagggagatcg	50.0%
9	tagaagatgggccttttcag	45.0%
10	acagaatgttttcccaaggg	45.0%
11	acctgtggatgaaagggaga	50.0%
12	tgtcatgtgcatcaacgtga	45.0%
13	aatgtttttgctagccactc	40.0%
14	ccagagtcgttaatgcatgt	45.0%
15	catatttatctgccaccatt	35.0%
16	aggcttaaatgctaacccat	40.0%
17	caggctacaaactcaggcaa	50.0%
18	gtaaaatctggtgccttcga	45.0%
19	tctttattcaggtgtatcca	35.0%
20	cccacgtgaacttgattcaa	45.0%
21	agtcatttatccatcagctc	40.0%
22	ttcagtacagcatgctcaat	40.0%
23	gaaagccagtatctgtatcc	45.0%
24	tccgtgaaacaggctacatt	45.0%
25	gaaggcagactgcaatgaca	50.0%
26	attgatccaggaagctgcag	50.0%
27	tgtatattgcaccaccttta	35.0%
28	atagttgagttggtgtcaca	40.0%
29	tttcacatttgtgacatgcc	40.0%
30	catcttagacccacaaggat	45.0%
31	acaaagaatgcctggtgtgg	50.0%
32	ccatcgttctgacaagtaca	45.0%
33	aggcgaaggtgatgaaacca	50.0%
34	agctatagaatgccttgttt	35.0%
35	acccacacaaggcttgaaaa	45.0%
36	tcaaagctgtcctttgaacc	45.0%
37	attcttcaagggaaccaact	40.0%
38	gaaaggcagaaggagaacct	50.0%
39	tatgagcaacacggcttcat	45.0%
40	cagctagtttcgaaccagat	45.0%
41	ccaagttctgagcgttagaa	45.0%
42	aacacccacacagtgtaaca	45.0%
43	gctgctccatgttataaact	40.0%
44	tctggttagccagaactatt	40.0%
45	cagactgttgacagagcgac	55.0%
46	tgcaaagtcatagtaggctc	45.0%
47	gggcagaagttcaccaagaa	50.0%
48	cacaacatctaccatcggtg	50.0%

Supplementary Table S4. PCR primers used in this study.

	**Amplicon**	**Left Primer**	**Right Primer**	**Experiment**
1	PCR1	CCAGTTCCGCCCATTCTCC	CTGTTATGCGGCCATTGTCC	Genomic DNA PCR
2	PCR2	TCGAGCAGACATGATAAGATAC	GTAACCGTGCATCTGCCAGT	Genomic DNA PCR
3	PCR3	TAGTTGCCAGCCATCTGTTG	GTAACCGTGCATCTGCCAGT	Genomic DNA PCR
4	MS2	GACCGCCTGAAGTCTCTGAT	TAGAAGGCACAGTCGAGG	Genomic DNA PCR
5	*Ccnb1* Promoter+*Luc*	CAACAAAGCTTTCGGGAACT	CCGGGCCTTTCTTTATGTTT	Genomic DNA PCR
6	*Ccnb1* EE	GCTCAGCAAGTTCCACCTCT	AGCGAAGAGCTACAGGCAAG	RT-qPCR
7	*Luciferase*	GAGGCGAACTGTGTGTGAGA	GTGTTCGTCTTCGTCCCAGT	ChIP and RT-qPCR
8	*NF-YA*	GTTAATGGTGCAAGTCAGTGGA	TCTGCTGTAAACCTTGTGTTCC	RT-qPCR
9	*Gapdh*	GCCAAAAGGGTCATCATCTC	CTAAGCAGTTGGTGGTGCAG	RT-qPCR
10	c-*Myc*	TGACCTAACTCGAGGAGGAGCTGGAATC	AAGTTTGAGGCAGTTAAAATTATGGCTG AAGC	RT-qPCR
11	*18S* rRNA	GTAACCCGTTGAACCCCATT	CCATCCAATCGGTAGTAGCG	RT-qPCR
12	*SV40* promoter	CAGTTCCGCCCATTCTCC	TCCTCACTACTTCTGGAATAGCTC	ChIP
13	*Hygromycin*	GGACCGATGGCTGTGTAGAA	CGAAGCCCAACCTTTCATAG	ChIP
14	*No Promoter:: Luc-MS2* Luciferase 5’-end	ATGTACGGGCCAGATATTCG	CCGGGCCTTTCTTTATGTTT	ChIP
15	*Ccnb1::Luc-MS2* transgene promoter	GAGCGAGTGCCACGAACT	CCGGGCCTTTCTTTATGTTT	ChIP
16	Chr. 19 intergenic region	AACCATCATTCCAAGCGGGT	GGATGAGATGGGCAAAACGC	ChIP
17	Native *Ccnb1* promoter	TAAACCTAAGCCCGGCAGAC	CCCGATTCGAGAAGACACC	ChIP
18	*Ccnb1* Exon 2	TGTGACAGTTACTGCTGCTTCC	GCCTGTAGCTCTTCGCTGAC	ChIP
19	*Ccnb1* intergenic region (Chr.13)	GACTCCACCACTGGGTCTGT	CTTCCCTCTTCCCTCCGTA	ChIP

Supplementary Table S5. Oligonucleotides used in splinkerette PCR.

**Name**	**Sequence**	**Experiment**
SPLNK-GATC-TOP	GATCCCACTAGTGTCGACACCAGTCTCTAATTTTTTTTTTCAAAAAAA	Generate annealed splinkerette adaptors
SPLNK-BOT	CGAAGAGTAACCGTTGCTAGGAGAGACCGTGGCTGAATGAGACTGGTGTCGACACTAGTGG	Generate annealed splinkerette adaptors
spPCR-Us1R	CCAGTGCTGCAATGATACCGCGAGAC	Splinkerette PCR
spPCR-Us2R	CACCGGCTCCAGATTTATCAGC	Splinkerette PCR
SPLNK #1	CGAAGAGTAACCGTTGCTAGGAGAGACC	Splinkerette PCR
UsSeq	AAGTGGTCCTGCAACTTTATCCG	Sequence PCR products
